# A timer for analyzing temporally dynamic changes in transcription during differentiation in vivo

**DOI:** 10.1083/jcb.201711048

**Published:** 2018-08-06

**Authors:** David Bending, Paz Prieto Martín, Alina Paduraru, Catherine Ducker, Erik Marzaganov, Marie Laviron, Satsuki Kitano, Hitoshi Miyachi, Tessa Crompton, Masahiro Ono

**Affiliations:** 1 Department of Life Sciences, Faculty of Natural Sciences, Imperial College London, London, England, UK; 2 Institute for Viral Research, Kyoto University, Kyoto, Japan; 3 University College London Great Ormond Street Institute of Child Health, London, England, UK

## Abstract

Understanding the mechanisms of cellular differentiation is challenging because differentiation is initiated by signaling pathways that drive temporally dynamic processes, which are difficult to analyze in vivo. We establish a new tool, Timer of cell kinetics and activity (Tocky; or toki [time in Japanese]). Tocky uses the fluorescent Timer protein, which spontaneously shifts its emission spectrum from blue to red, in combination with computer algorithms to reveal the dynamics of differentiation in vivo. Using a transcriptional target of T cell receptor (TCR) signaling, we establish *Nr4a3*-Tocky to follow downstream effects of TCR signaling. *Nr4a3*-Tocky reveals the temporal sequence of events during regulatory T cell (Treg) differentiation and shows that persistent TCR signals occur during Treg generation. Remarkably, antigen-specific T cells at the site of autoimmune inflammation also show persistent TCR signaling. In addition, by generating Foxp3-Tocky, we reveal the in vivo dynamics of demethylation of the *Foxp3* gene. Thus, Tocky is a tool for cell biologists to address previously inaccessible questions by directly revealing dynamic processes in vivo.

## Abstract (precis)

Bending et al. establish a new tool, Timer of cell kinetics and activity (Tocky), revealing the temporal dynamics of cellular activation and differentiation in vivo. The tool analyzes the temporal sequence of molecular processes during cellular differentiation and can classify cells based on the frequency they receive signaling events in vivo.

## Introduction

It is a central question in cell biology how cellular differentiation progressively occurs through the activities of temporally coordinated molecular mechanisms (Kohwi and Doe, 2013; Kurd and Robey, 2016). It is, however, challenging to investigate in vivo mechanisms at the single-cell level because individual cells are not synchronized and are heterogeneous, receiving key signaling at different times and frequencies in the body. No existing technologies can systematically analyze the temporal dynamics of differentiation and activities of individual cells in vivo. Intravital microscopy is useful for analyzing cells in microenvironments (Koechlein et al., 2016) but is not suitable for systematically analyzing cells that rapidly migrate through tissues such as T cells. Single-cell sequencing can provide “pseudotime,” but this is not the measurement of time as the name implies; rather, it is a measurement of the transcriptional similarities between samples at chosen analysis time points (Trapnell et al., 2014). Flow cytometry is suitable for determining the differentiation stage of individual cells, but current methods cannot be applied to investigate how individual cells sequentially differentiate into more mature stages as data from individual cells do not currently encode time information (Hoppe et al., 2014). There is thus a great need for a new technology to experimentally analyze the passage of time after a key differentiation event, or the time domain, of individual cells in vivo. Such a new technology would benefit all areas of cellular biology, but it would be particularly useful for the study of T cells under physiological conditions in vivo, where both the time and frequency of signaling are critical to their differentiation.

T cells migrate through the body (Krummel et al., 2016), and their activation and differentiation statuses are almost exclusively determined by flow cytometric analysis (Fujii et al., 2016). In T cells, T cell receptor (TCR) signaling triggers their activation and differentiation (Cantrell, 2015) and is the central determinant of thymic T cell selection (Kurd and Robey, 2016), including negative selection (Stepanek et al., 2014) and regulatory T (Treg) cell selection (Picca et al., 2006) and antigen recognition in the periphery (Cantrell, 2015). Although the temporal dynamics of proximal TCR signaling, which are in the timescale of seconds, have been comprehensively and quantitatively analyzed (Roncagalli et al., 2014; Stepanek et al., 2014), it is still unclear how transcriptional mechanisms for activation and differentiation respond to TCR signals over time in vivo. Such a transcriptional mechanism may be used for a new reporter system to analyze the dynamics of T cell activation and differentiation upon antigen recognition.

TCR signaling activates NFAT, AP-1, and NF-κB, which initiate the transcription of immediate early genes within a few hours (Oh and Ghosh, 2013), but their effects on T cell differentiation over the timescale of hours and days are obscure. To analyze TCR signal strength, currently, *Nur77 (Nr4a1)-EGFP* reporter mouse is commonly used (Moran et al., 2011), but the long half-life of the reporter gene EGFP (∼56 h; Sacchetti et al., 2001) prevents its application for the analysis of the temporal dynamics of the events downstream of TCR signaling in vivo.

In this study, we have established a new fluorescent Timer technology, Timer of cell kinetics and activity (Tocky; toki means time in Japanese), which uniquely reveals the time and frequency domains of cellular differentiation and function in vivo. Fluorescent Timer proteins have been used to analyze in vivo protein dynamics and receptor turnover (Khmelinskii et al., 2012; Donà et al., 2013) as well as identify progenitor cells (i.e., those cells expressing only immature fluorescence during embryogenesis and pancreatic β cell development; Terskikh et al., 2000; Subach et al., 2009; Miyatsuka et al., 2011, 2014). However, those studies were qualitative and did not recognize the quantitative power of fluorescent Timer. In this study, we develop a new fluorescent Timer approach to quantitatively analyze the time and frequency domains of gene transcription within individual cells in vivo. By identifying a downstream gene of TCR signaling (*Nr4a3*) and developing fluorescent Timer reporter mice for the gene, we experimentally establish and validate the Tocky system for TCR signaling. Furthermore, we apply the Tocky approach to the *Foxp3* gene, which is the lineage-specific transcription factor of Treg cells, revealing in vivo dynamics of Treg cell differentiation. Thus, Tocky technology reveals time-dependent mechanisms of in vivo cellular differentiation and developmental states after key signaling pathway or lineage commitment, which cannot be analyzed by existing technologies.

## Results

### Design of the Tocky system for analyzing the time and frequency domains of signal-triggered activation and differentiation events

**Figure 1.**
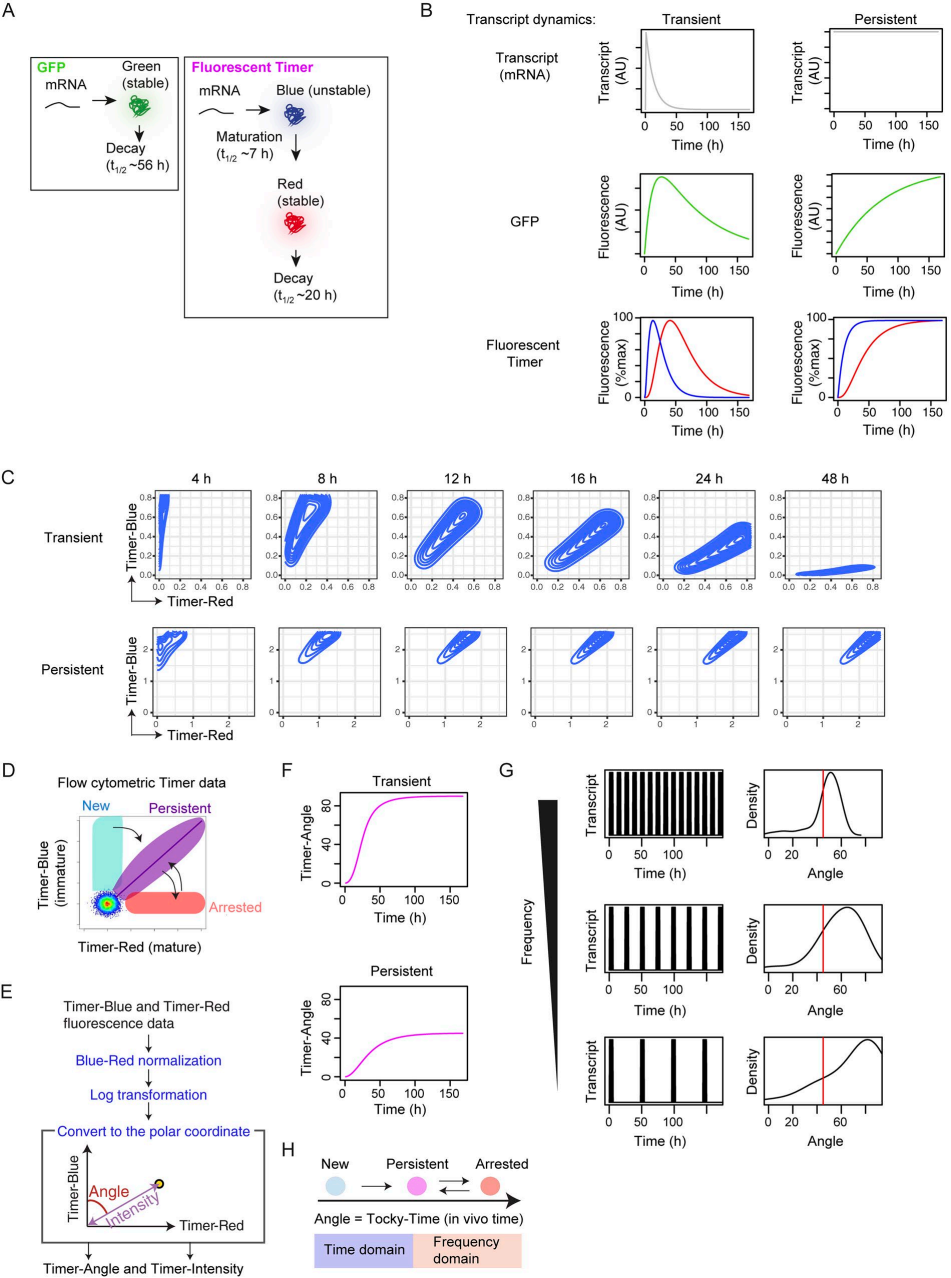
**Design of Tocky system for analyzing the time and frequency domains of signal-triggered activation and differentiation events. (A)** Production and decay of GFP and fluorescent Timer protein. **(B)** In silico analysis of GFP and Timer fluorescence by different transcriptional dynamics. Time-course analysis of GFP or blue and red forms of Timer protein, simulated flow cytometric data, and Timer Angle, given the constant influx of cells with the indicated transcriptional dynamics. Data from spike-like (transient) and constant transcriptional dynamics are shown. **(C)** In silico modeling of flow cytometric data depicting transient (top) or persistent Timer transcription (bottom). **(D)** Timer locus approach to translate flow cytometric Timer fluorescence data into transcriptional dynamics. **(E)** Schematic representation of trigonometric data transformation of flow cytometric Timer data. Flow cytometric blue and red Timer fluorescence data were preprocessed and normalized and subsequently transformed by a trigonometric function. **(F)** Effect of persistent or transient transcription on Timer Angle progression. **(G)** In silico modeling of the effect of transcriptional frequency on the distribution of Timer Angle values. **(H)** Model for analyzing the time domain and frequency domain using Tocky time analysis.

Given the long half-life of stable fluorescent proteins (FPs) like GFP (56 h; Sacchetti et al., 2001), the dynamics of gene transcription cannot be effectively captured using conventional FP expression as a reporter. We therefore chose to use fluorescent Timer protein (Timer), which forms a short-lived chromophore that emits blue fluorescence (blue), before producing the mature chromophore that emits red fluorescence (red). The maturation half-life (i.e., the production of red-form proteins) is estimated to be 7 h, whereas red proteins are stable and have a decay rate >20 h (Fig. 1 A; Subach et al., 2009).

We investigated in silico how two different transcriptional activities would influence the production of blue and red fluorescent form of the protein through time, and how the light emitted would be detected by flow cytometry. We compared the reporting of transient, pulse-like transcription with that of persistent transcription, both of which are relevant to TCR-mediated transcription (Fig. 1 B; Yosef and Regev, 2011). A linear kinetics model showed that blue was a better readout for the real-time level of transcription than red and GFP, which rather reported the cumulative activity of transcription. Next, we analyzed the dynamics of Timer-expressing cells in the blue–red plane, which is relevant for flow cytometric analysis. Assuming that cells received a transient signal in a synchronized manner, cells showed a fan-like movement from blue^+^red^−^ to blue^−^red^+^, and cells stayed until the red-form proteins decayed. However, when cells receive persistent TCR signals, cells gradually approached the diagonal line between blue and red axes, which is the steady state (Fig. 1 C). Thus, there are three key loci in the blue–red plane (Fig. 1 D). First, when new transcription occurs in Timer-negative cells, Timer-positive cells acquire pure blue and are identified in the New locus. Second, if transcriptional activity is persistent and/or sufficiently frequent, then cells accumulate in and around the steady-state diagonal line (Persistent locus). Lastly, when transcription is diminished, cells lose blue and stay in the Arrested locus until the red protein decays (Fig. 1 D). Importantly, Timer maturation is unidirectional and irreversible as the chromophore matures from blue to red. In the case of individual Timer-positive cells, movement from New to Persistent loci is also unidirectional and irreversible because the half-life of red protein is longer than the half-life of the blue form, whether the signal is transient or continuous (Fig. 1 C). Therefore transition from the New to the Persistent loci captures the time domain of cellular differentiation. In contrast, cells in the Arrested locus may reinitiate transcription to express new blue protein and move anticlockwise back into the Persistent locus (Fig. 1 D). This leads to the hypothesis that the movement between Persistent and Arrested loci more specifically captures how frequently transcriptional activities occur in mature cells.

These three loci can be identified and quantified more effectively by analysis of the angle of individual cells from the blue axis. The 2D blue versus red Timer fluorescence data can be transformed by trigonometric data transformation and converted into the polar coordinate to provide new variables: the angle from the blue axis is defined as Timer Angle, which is a measure of the trajectory and change in transcriptional history. The Euclid distance from the origin is defined as Timer Intensity (Fig. 1 E) and is a measure of the signal strength given Timer Angle value.

In fact, trigonometric data transformation quantitatively showed that transient signals had a faster progression of Timer Angle than continuous signals (Fig. 1 F). Next, using Timer Angle, we analyzed transcriptional activities with different frequencies. As implicated by the analysis of transient and continuous signals (Fig. 1, B and C), when the frequency of transcription was low, cells accumulate in higher Timer Angle. In contrast, if cells receive high-frequency signals, they approach the steady state of continuous transcription (Fig. 1 G).

We therefore coined the system Tocky. Timer Angle provides time-related composite information: the first phase (New → Persistent) is for analyzing how differentiation mechanisms are regulated over time (i.e., time domain analysis); and the latter half (Persistent – Arrested) is for analyzing the relative frequency of transcriptional activation (i.e., frequency domain analysis). Thus, we designate this composite time axis as Tocky time (Fig. 1 H). In Time domain analysis in physics and related subjects, data obtained over time are transformed into frequency data (and vice versa) using a function such as the Fourier transform. In contrast, the Tocky time can be the measurement of either time or frequency, depending on the Timer maturation phase. Notably, these two domains merge in the Tocky time when transcriptional activities are persistent.

### The development of *Nr4a3*-Tocky for the analysis of TCR signal downstream events

**Figure 2.**
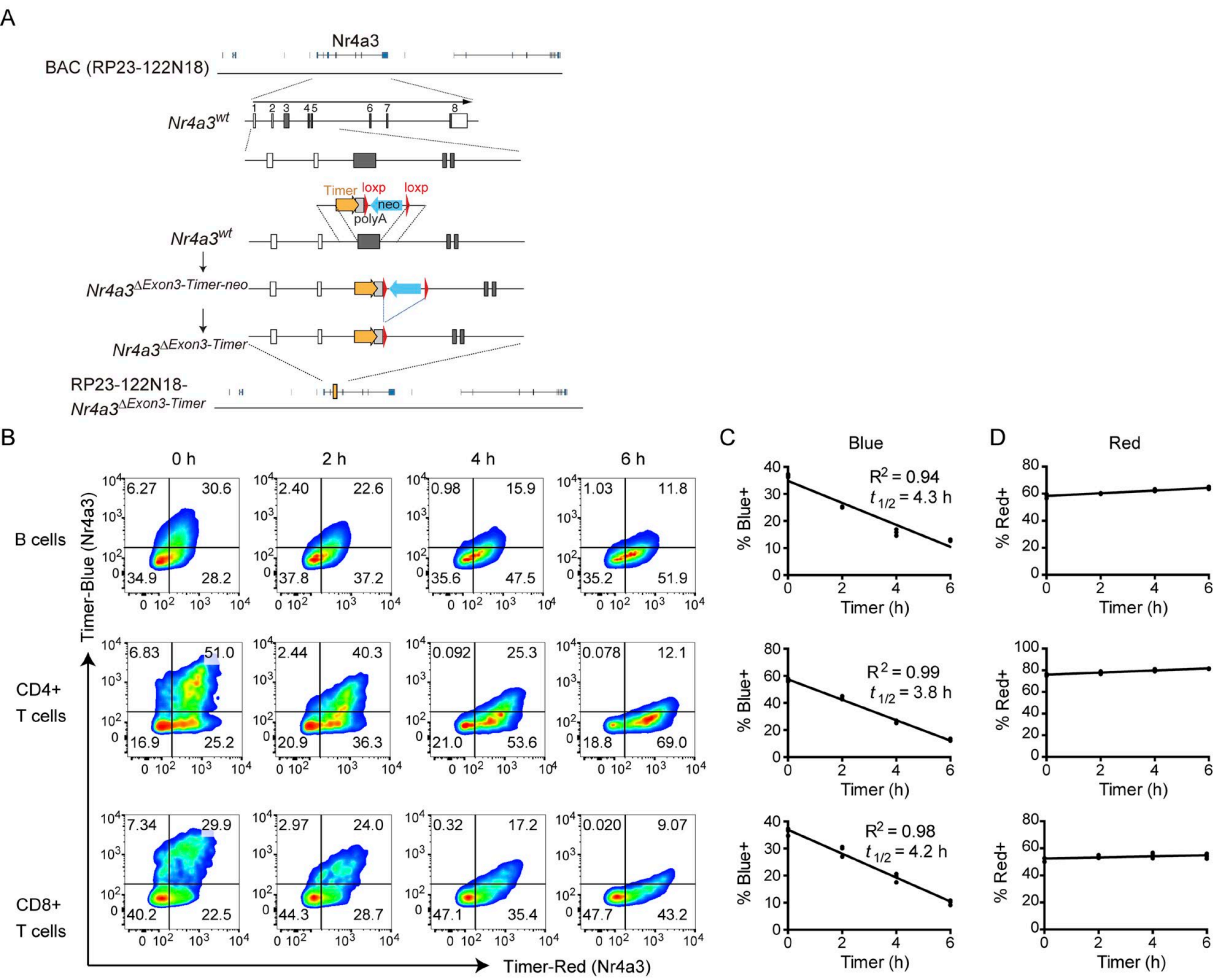
**Antigen–receptor ligation induces Timer proteins in B and T cell subsets from *Nr4a3*-Tocky mice. (A)** Construct for generating *Nr4a3*-Tocky BAC transgenic mice. **(B)** Splenocytes from *Nr4a3*-Tocky mice were activated for 20 h with either 10 µg/ml of soluble goat anti–mouse IgM (for CD19^+^ B cells) or 2 µg/ml plate bound anti-CD3 (for CD4^+^ and CD8^+^ T cells). Cells were then incubated with 100 μg/ml cycloheximide to inhibit new protein translation and the decay of blue fluorescence measured over time by flow cytometry. Shown is Timer–blue versus Timer–red fluorescence in CD19^+^ B cells (top), CD4^+^ T cells (middle), or CD8^+^ T cells (bottom) at the indicated time points. **(C and D)** Summary data of the percentage of cells blue^+^ (C) or red^+^ (D) cells in the cultures. Linear regression by Pearson’s correlation; *n* = 3 culture triplicates. Data are representative of two independent experiments.

T cell differentiation is triggered by TCR signals. To track the in vivo dynamics of transcription downstream of TCR signal transduction, we first identified genes immediately downstream of TCR signaling using a data-oriented multidimensional analysis, canonical correspondence analysis (CCA; Ono et al., 2014; Fig. S1 A). *Nr4a3* (*Nor1*) was identified as the gene with the highest correlation with anti-CD3–mediated T cell activation (which mimics TCR activation) and in vivo TCR signals in the thymus, whereas *Nr4a1* (*Nur77)* and *Rel* also scored highly (Fig. S1, B and C). In agreement, upon anti-CD3 stimulation, *Nr4a3/Nor1* was rapidly induced in T cells and peaked within 2 h of stimulation (Fig. S1 D). Having established in silico that Tocky can temporally report transcription, we generated a bacterial artificial chromosome (BAC) transgenic reporter *Nr4a3-*Tocky mice (Fig. 2 A), in which the transcriptional activity of the *Nr4a3* gene is reported by Timer proteins and used as an indicator of new transcription mediated by TCR signal transduction.

### Antigen–receptor ligation induces Timer proteins in B and T cell subsets from *Nr4a3*-Tocky mice

Using *Nr4a3*-Tocky, antigen–receptor ligation induced Timer protein expression not only in CD4^+^ T cells but also in CD8^+^ T cells and B cells, with the most prominent induction in CD4^+^ T cells (Fig. 2 B). This provided us an opportunity to experimentally determine the half-life of the blue fluorescent form of Timer protein in each cell subtype and thereby ask whether the kinetics of blue-expressing cells are similar between different cell subtypes. B and T cells were stimulated by antigen–receptor ligation and immediately after the induction of Timer proteins, protein translation was blocked by cycloheximide and the decay of blue fluorescence was measured by flow cytometry (Fig. 2 C). Linear regression analysis showed that the half-life of blue-expressing cells was ∼4 h in all of the cell subtypes, whereas the percentage of red-expressing cells was barely changed or slightly increased after the conversion of blue proteins into red proteins (Fig. 2 D). These data support that Timer maturation is not affected by cell subtypes.

### *Nr4a3*-Tocky reveals the temporal dynamics and frequency of TCR signal-triggered activation events

**Figure 3.**
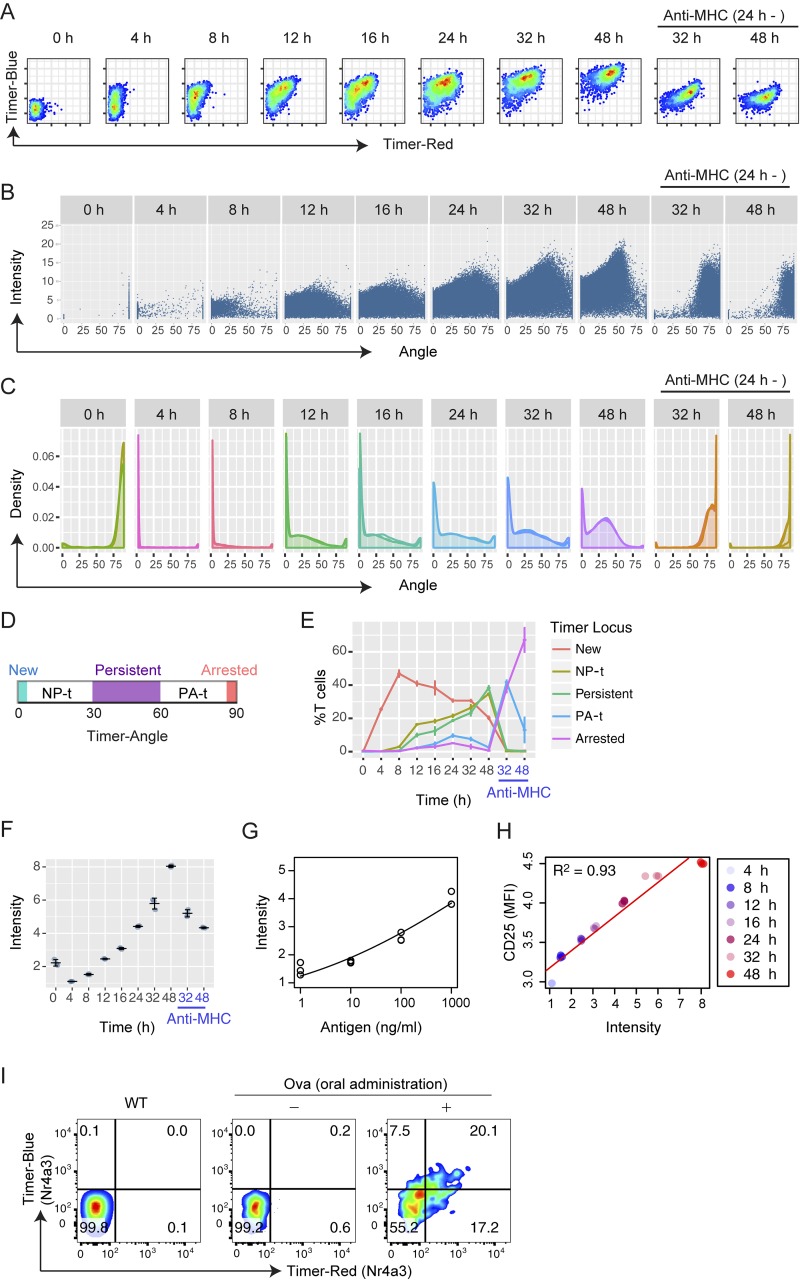
***Nr4a3*-Tocky reveals the time domain of TCR signal–triggered activation and differentiation events. (A)** Flow cytometric analysis of blue and red Timer raw fluorescence in antigen-stimulated OT-II *Nr4a3-*Tocky T cells. T cells were stimulated for the indicated time points with 1 µM Ova peptide. In some cultures, anti–MHC II antibodies were added at 24 h to terminate TCR signaling. **(B)** Trigonometric-transformed data from A. Individual cells are plotted against Timer Angle and Timer Intensity. **(C)** Density plot of Timer Angle from the transformed data. **(D)** The designation of the five Timer loci by Timer Angle θ as follows: New (θ = 0°), NP-t (0° < θ < 30°), Persistent (30° ≤ θ < 60°), PA-t (60° ≤ θ < 90°), and Arrested (θ = 90°). **(E)** Timer Locus analysis to show the frequency of cells within the five Timer loci defined in C. **(F)** Summary of Timer Intensity in the cultures from A over time. **(G)** Dose–response curve of Timer Intensity on stimulation with titrated doses of Ova peptide (antigen). OT-II *Nr4a3-*Tocky T cells were stimulated for 22 h in the presence of 1, 10, 100, or 1,000 nM Ova peptide and APCs. Data were fitted to a dose–response curve with a statistical significance by a lack-of-fit test (P = 0.014). **(H)** Scatter plot of Timer Intensity versus cell surface CD25 expression. Linear regression analysis showed a strong correlation (R^2^ = 0.93). See legend for sample identities. **(I)** CD4^+^ T cells from OT-II TCR transgenic *Nr4a3*-Tocky mice were adoptively transferred in to CD45.1 congenic mice, which were fed Ova or control for 3 d. CD45.2^+^CD45.1^+^CD4^+^ OT-II *Nr4a3*-Tocky T cells within mesenteric lymph nodes were analyzed for Timer expression. Error bars represent mean ± SD; *n* = 3 culture triplicates. Data are representative of at least two independent experiments.

To analyze the T cell response upon antigen recognition, we generated OT-II *Nr4a3-*Tocky mice, which express ovalbumin (Ova)-specific transgenic TCR. Ova stimulation of CD4^+^ T cells from OT-II *Nr4a3-*Tocky mice resulted in the up-regulation of blue within 4 h, with some cells acquiring red at 8 h after stimulation (Fig. 3 A). *Nr4a3-*Tocky T cells further increased both blue and red throughout the 48-h culture, whereas removal of TCR stimulation by anti–major histocompatibility complex (MHC) II treatment from 24 h onwards resulted in the rapid loss of blue (Fig. 3 A), which captured the reduction in frequency of TCR signaling.

To analyze effectively the continuous progression of Timer fluorescence in Fig. 3 A, Timer fluorescence data were transformed into Angle and Intensity data (Fig. 3 B). This transformation of the data normalizes for blue and red fluorescence by using the mean and SD of the negative cloud of cells (i.e., cells with autofluorescence only). To generate robust angle values, thresholding of the data are required to restrain angles between 0° and 90°. This thresholding sets the blue and red levels for positivity and thus collapses pure blue and pure red cells into angles of 0° and 90°, respectively (see Materials and methods).

Importantly, the progression of Timer Angle becomes slower and cells accumulate as cells approach ∼45° by density plots, which visualize the distribution of angle values, reflecting sustained or high-frequency TCR signaling (Fig. 3 C). To quantify the maturation of the Timer chromophore since first onset of its translation, we grouped cells into five populations (i.e., data categorization) according to their Angle values: from the “New” population (Angle = 0°) representing cells within the first 4 h of initiation of TCR-mediated transcription to the “Arrested” population (Angle = 90°), which represents cells in which all Timer protein has matured to red and transcriptional activity has decreased below cytometer detection thresholds (Fig. 3 D). The area between 30° and 60° was defined as the Persistent locus. The areas between New and Persistent (NP-t) or between the Persistent and Arrested (PA-t) contained cells from the surrounding loci that are in the process of changing their frequency of *Nr4a3* transcription (Fig. 3 E). The analysis of the percentage of cells in these loci (designated as Timer locus analysis) neatly captured the change in TCR-mediated transcription through time as cells shifted from New to Persistent, which represent the time domain. Furthermore, it showed that removal of TCR signals lead to a complete loss of New, NP-t, and Persistent signaling within 8 h, and cells migrated to Arrested transcriptional dynamics, which represents sparse or no signaling activity (Fig. 3 E). Thus, *Nr4a3*-Tocky recaptured the predicted kinetics of Timer-expressing cells, validating the Tocky model (Fig. 1 H).

We hypothesized that Timer Intensity reflects both the signal strength and the duration/frequency of TCR signaling as Timer proteins accumulate in individual cells in response to strong and/or repeated TCR signals. Timer Intensity in antigen-stimulated *Nr4a3-*Tocky T cells increased over time and fell after removal of the TCR signal (Fig. 3 F). Furthermore, Timer Intensity was increased in a dose-dependent manner by cognate antigen (Fig. 3 G). Interestingly, Timer Intensity showed a high correlation to cell surface CD25 expression, which is a marker of activated T cells (R^2^ = 0.93; Fig. 3 H). Thus, using *Nr4a3-*Tocky, Timer Intensity reflects the cumulative transcriptional outcome of signals in a given cell as the reporter protein accumulates in response to sustained or highly frequent signals.

Next, we addressed whether *Nr4a3*-Tocky identifies T cells that receive TCR signals in vivo. We adoptively transferred OT-II TCR transgenic *Nr4a3*-Tocky T cells into congenic recipients, which were then fed their cognate antigen, Ova, in the water (or water alone; Fig. 3 I). This model allows analysis of T cell responses in mesenteric lymph nodes to orally administered antigens. Timer expression occurred in OT-II TCR transgenic T cells from *Nr4a3*-Tocky mice only in the presence of their cognate antigen, indicating that Timer expression is induced upon antigen recognition in vivo.

**Figure 4.**
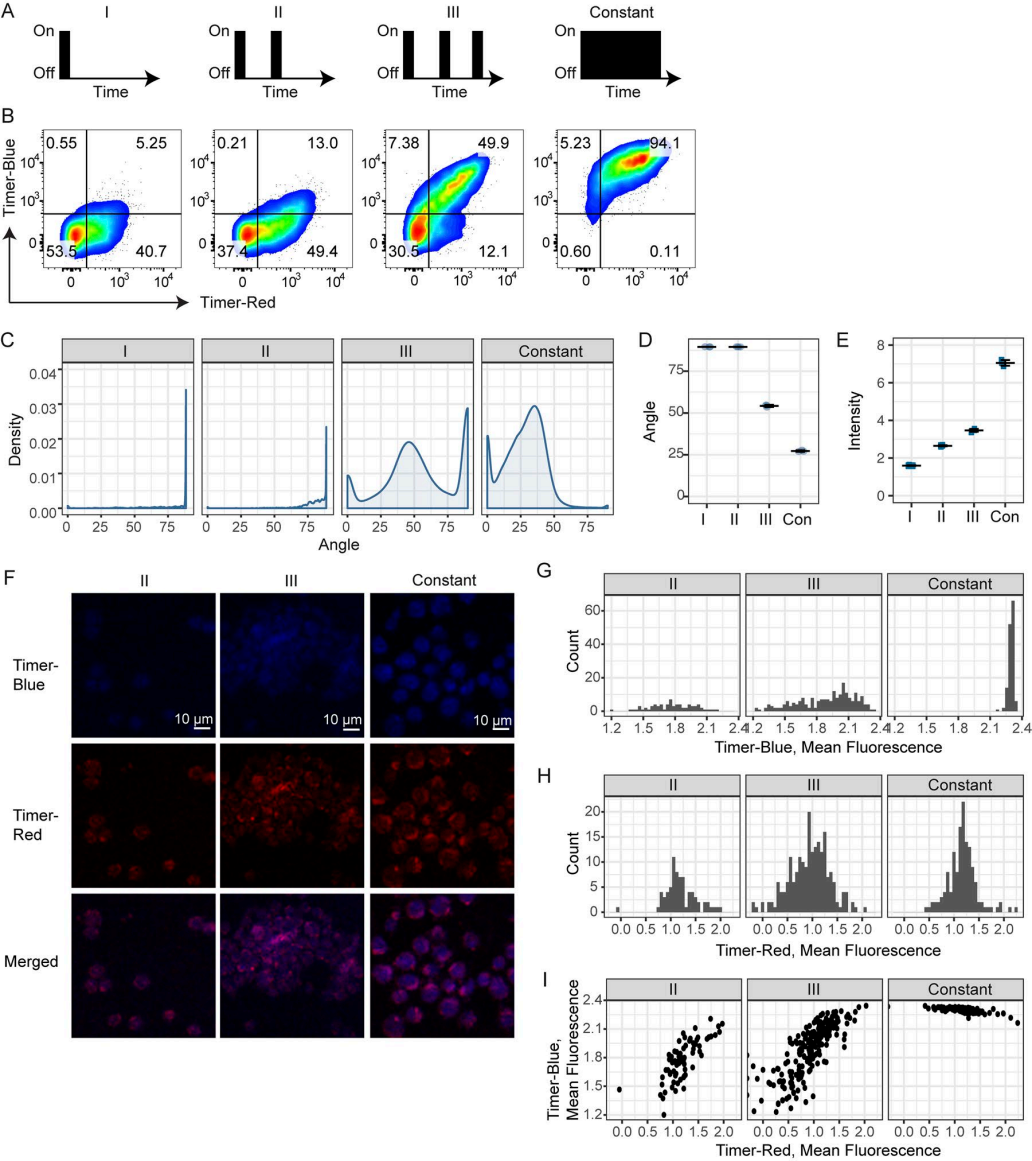
***Nr4a3*-Tocky captures the frequency domain of TCR signaling. (A–I)** T cells from OTII *Nr4a3*-Tocky mice were cultured with T cell–depleted splenocytes. **(A)** T cells were stimulated with 1 µM Ova as follows: 4-h stimulation, then rested for 48 h (I); 4-h stimulation, 20-h rest, 4-h stimulation, and 24-h rest (II); 4-h stimulation, 20-h rest, 4-h stimulation, 20-h rest, and 4-h stimulation (III); or 2-d constant (Con) stimulation. During resting stages, cells were incubated with 100 U/ml recombinant IL-2 and 20 µg/ml anti–MHC II and washed before and after stimulations. **(B)** Cells were then harvested and CD4^+^ OTII T cells analyzed for Timer–blue versus Timer–red fluorescence expression by flow cytometry. **(C)** Displayed are the Timer Angle distributions for the four culture conditions. **(D and E)** Shown are the mean Timer Angle (D) or Timer Intensity (E) within the four different cultures. Error bars represent mean ± SD; *n* = 3 culture triplicates. **(F)** Confocal microscopy analysis of OTII *Nr4a3*-Tocky stimulated with various frequencies. The same cell samples used in A–D were analyzed by confocal microscopy. Bars, 10 µm. **(G and H)** Histogram showing the mean fluorescence intensity of Timer–blue (G) or Timer–red fluorescence (H) in single cells by microscopic image analysis. **(I)** 2D plots of the mean fluorescence intensities of Timer–blue and Timer–red fluorescence in single cells by microscopic image analysis. Cells were identified as regions of interest, and mean fluorescence intensities were measured and logged. See Fig. S2 and Materials and methods for details.

To validate the frequency domain of the Tocky system, we used the *Nr4a3*-Tocky OTII system to perform periodic stimulation of T cells with cognate peptide. T cells underwent one, two, or three rounds of 4-h peptide stimulation over a 48-h period (the groups I, II, and III), and the Timer blue versus red expression was analyzed by flow cytometry and compared with constant stimulation (Constant; Fig. 4 A). As predicted, low frequency stimulation resulted in predominantly pure red expression (Fig. 4 B). With increased frequency of signaling, blue fluorescence increased, which was highest in cells undergoing constant stimulation. Analysis of Timer Angle distribution captured the shift in frequency as Angles moved from the Arrested locus (i.e., 90°) toward the Persistent locus with increasing frequency of stimulus (Fig. 4, C and D). As expected, Timer Intensity also showed a frequency dependent relationship as more Timer proteins accumulated in response to more frequent TCR stimulation (Fig. 4 E).

Next, we have further analyzed the same stimulated T cells using confocal microscopy analysis (Fig. 4, F–I; and Fig. S2, A and B). Visual inspections of microscopic images suggested that Timer–blue fluorescence was increased as the frequency of stimulation increased (Figs. 4 F and S2 A). Quantitative measurement of fluorescent intensities in individual cells confirmed this (Fig. 4, G–I; and Fig. S2 B). Both Timer–blue and Timer–red fluorescence increased as the stimulation was more frequently applied, and the increase was more remarkable in Timer–blue fluorescence (Fig. 4, G and H). 2D plot of Timer–blue and Timer–red fluorescence by confocal microscopy (Fig. 4 I) recaptured the flow cytometric result (Fig. 4 B). Thus, this single-cell microscopy experiment further validated the Tocky system.

### Cell division, costimulation, and IL-2 signaling do not affect Timer Angle progression

**Figure 5.**
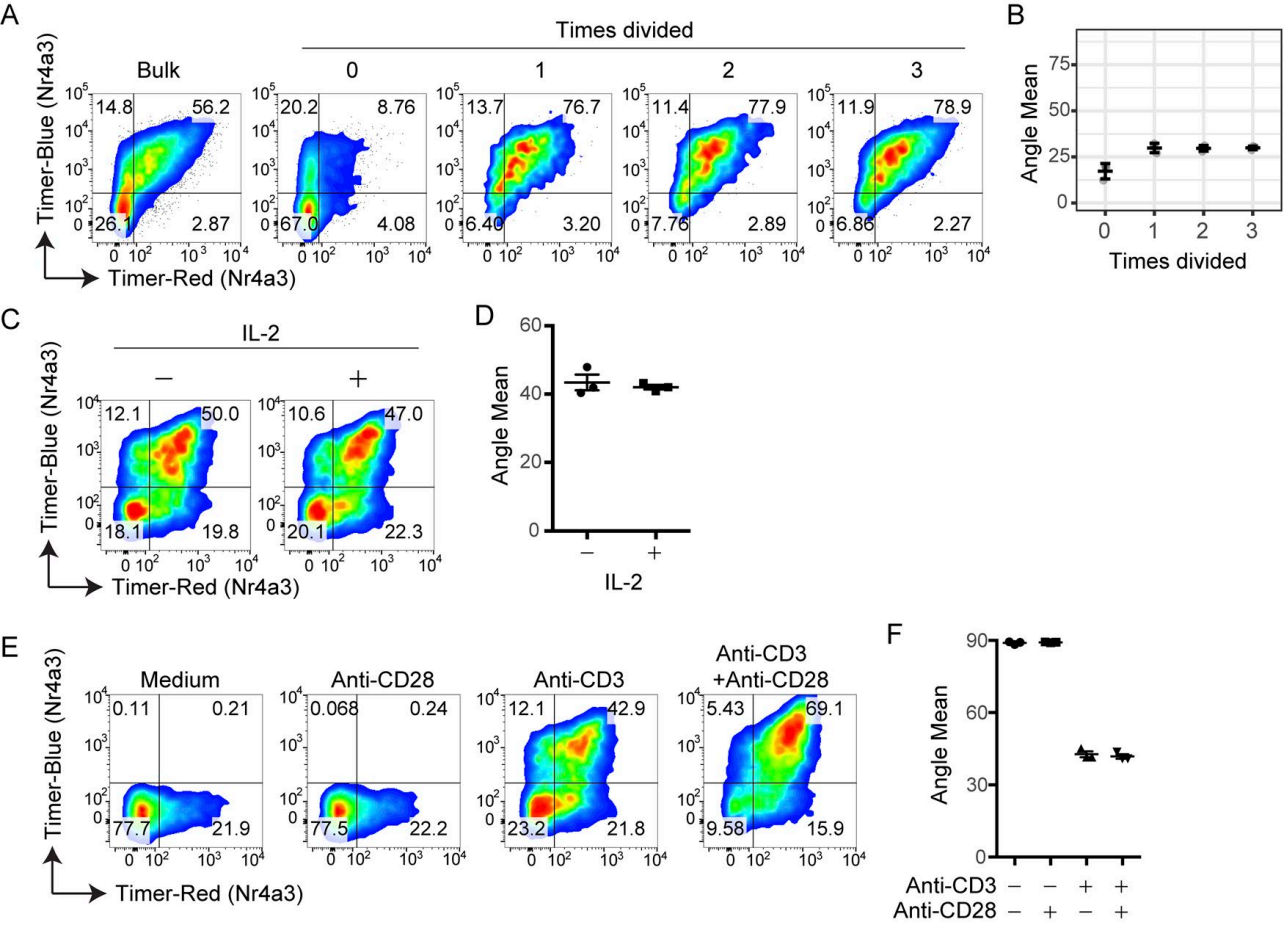
**Cell division, costimulation, and IL-2 signaling do not affect Timer Angle progression. (A–F)** CD4^+^ T cells from *Nr4a3*-Tocky mice were labeled with a proliferation dye and activated for 72 h with anti-CD3. Cells were then analyzed based on dilution of proliferation dye and classified into number of cellular divisions. **(A)** Timer–blue versus Timer–red fluorescence in CD4^+^ T cells gated on dilution of proliferation dye. **(B)** Mean Timer Angle values in the cultures from A. **(C and D)** Splenocytes from *Nr4a3*-Tocky mice were stimulated on anti-CD3–coated plates in the presence or absence of 100 U/ml rhIL-2 for 20 h. Timer–blue versus Timer–red fluorescence in CD4^+^ T cells from cultures (C). Mean Timer-Angle in cultures (D). **(E and F)** Splenocytes from *Nr4a3*-Tocky mice were stimulated on plates coated with anti-CD28 alone, anti-CD3 alone, or anti-CD3 + anti-CD28 for 20 h. Timer–blue versus Timer–red fluorescence in CD4^+^ T cells from cultures (E). Mean Timer-Angle in cultures (F). *n* = 3 culture triplicates; error bars represent mean ± SEM. Data are representative of two independent experiments.

Next, we examined whether processes related to T cell activation affect Timer Angle. TCR signaling leads to T cell activation and proliferation. Because each cell division halves both existing blue- and red-form proteins, it was predicted that cell division would not change Timer Angle. In fact, by analyzing the dilution of a proliferation dye as cells divide, activated T cells did not change their Timer Angle after cell division (Fig. 5, A and B). Activated T cells produce IL-2, which promotes the survival and proliferation of these T cells (Hoyer et al., 2008). Exogenous IL-2 had no effect on Timer Angle (Fig. 5, C and D). CD28 signaling enhances the activities of TCR signal downstream, inhibiting apoptosis and sustaining the activation processes (Boomer and Green, 2010; Walker and Sansom, 2011). Importantly, anti–CD28 antibody alone did not induce Timer expression, and it also did not change the progression of Timer Angle by TCR signals (Fig. 5, E and F). Collectively, the data above indicate that the progression of Timer Angle is not affected by cell division or activation status but is defined by the time and signal dynamics and that *Nr4a3*-Tocky reports the temporal dynamics of TCR signal downstream activities.

### The time-domain analysis of *Nr4a3*-Tocky mice delineates the temporal sequences of thymic Treg cell differentiation in vivo

Having validated the *Nr4a3*-Tocky system, we decided to investigate thymic Treg cell differentiation. TCR signaling is the major determinant of Treg cell differentiation in the thymus. T cells that have recognized their cognate antigens and received strong TCR signals preferentially express CD25 and Foxp3 and differentiate into Treg cells (Hsieh et al., 2012; Weissler and Caton, 2014).

**Figure 6.**
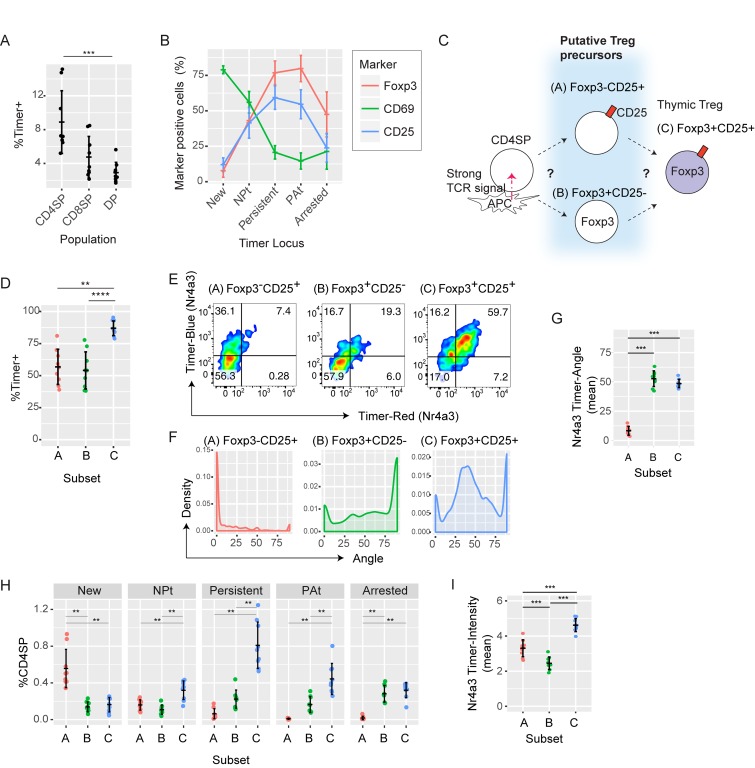
***Nr4a3****-***Tocky mice identify thymic Treg cell precursors. (A)** Percentages of Timer-positive cells in the indicated thymic T cell populations from *Nr4a3-*Tocky mice. DP, double positive. **(B)** Mean percentages of CD4SP cells expressing CD69 (green), CD25 (blue), or Foxp3 (red) from the five Timer loci. **(C)** Current working model for thymic Treg cell differentiation. **(D)** Percentages of Timer^+^ cells in the indicated CD4SP subpopulations (A, CD25^+^Foxp3^−^; B, CD25^−^Foxp3^+^; and C, CD25^+^Foxp3^+^). **(E)** Timer blue and red fluorescence from CD4SP Thymic Treg cell subsets from 7-d-old neonates. **(F)** Density plot of Timer Angle from the transformed data. **(G)** The mean Timer Angle of thymic Treg cell subsets. **(H)** Frequency within the CD4SP thymic population of each Timer locus in the three Treg cell subsets. **(I)** The mean Timer Intensity of thymic Treg cell subsets. Thymi from 7-, 9-, and 13-d-old neonates were analyzed in three independent experiments, and data were combined unless otherwise indicated. Error bars represent mean ± SD. **, P < 0.01; ***, P < 0.001; ****, P < 0.0001.

To demonstrate the quantitative power of the Tocky technology, we investigated the temporal sequence of thymic CD4 T cell differentiation after TCR signals by analyzing the time domain of ex vivo T cells from *Nr4a3-*Tocky thymus. Timer expression occurred in the CD4^+^CD8^+^ double-positive, CD4 single-positive (SP), and CD8SP populations, with CD4SP displaying the highest frequency (8.9 ± 3.7%; Fig. 6 A). The cell-surface expression of CD69, which is highly expressed on immature CD4SP, was high in CD4SP cells in the New locus and progressively declined as the Timer protein matures. In contrast, the majority of CD4SP cells in the Persistent locus expressed Foxp3 and CD25 (Fig. 6 B). With the assumption that in neonatal mice, nearly all cells in the Persistent locus would be derived from the New locus, these results led to the hypothesis that Treg cell differentiation requires persistent TCR stimulation.

The sequence of thymic Treg cell development is controversial. Some studies suggest that CD25^+^Foxp3^−^ Treg cells are the major Treg cell precursors (Burchill et al., 2007; Lio and Hsieh, 2008), whereas other research argues that CD25^−^Foxp3^+^ cells are Treg cell precursors (Fig. 6 C; Tai et al., 2013). These studies used in vitro culture experiments and intrathymic injection of precursor populations, which may not reflect the differentiation dynamics in vivo. We therefore revisited this issue to reveal the temporal sequences of thymic Treg cell differentiation in otherwise unmanipulated animals by analyzing the time domain of *Nr4a3*-Tocky.

The majority of Treg cells (CD25^+^Foxp3^+^) were Timer^+^ (∼90%), and both of the proposed Treg cell precursor populations (CD25^+^Foxp3^−^ and CD25^−^Foxp3^+^) also had high proportions of Timer^+^ cells (∼63% and ∼58%, respectively; Fig. 6 D), indicating that these three populations have all received either strong or frequent TCR signals. To place the three populations in time after TCR signal transduction, we analyzed thymi from *Nr4a3-*Tocky neonates and measured Foxp3 and CD25 expression in addition to Timer fluorescence. Timer expressing CD25^+^Foxp3^−^ cells were mostly blue^+^red^−^, whereas those of CD25^+^Foxp3^+^ Treg and CD25^−^Foxp3^+^ cells were mostly blue^+^red^+^ (Fig. 6 E). Most of CD25^+^Foxp3^−^ cells had the Angle value 0, whereas CD25^+^Foxp3^+^ Treg cells showed a clear peak ∼30°–50° and CD25^−^Foxp3^+^ cells had a higher peak at 90° (Fig. 6 F). The mean of Angle was not significantly different between CD25^+^Foxp3^+^ Treg cells and CD25^−^Foxp3^+^ cells (Fig. 6 G). These indicate that the CD25^+^Foxp3^−^ population is in the earliest time after receiving TCR signals. Next, to define the temporal relationships between the three Treg cells and precursor populations, we quantified the proportion of Timer^+^ cells in each maturation stage of Timer fluorescence (Fig. 6 H). This showed that most of the CD4SP cells in the New locus were CD25^+^Foxp3^−^ cells, whereas most of CD4SP cells in the Persistent were CD25^+^Foxp3^+^ Treg cells. The relative contribution of CD25^−^Foxp3^+^ was greatest in the Arrested locus, suggesting that these cells are enriched with those with aborted TCR signaling (Fig. 6 H). In addition, Timer intensity analysis showed that the CD25^−^Foxp3^+^ subset had received the weakest and/or least frequent TCR signals among these subsets (Fig. 6 I).

**Figure 7.**
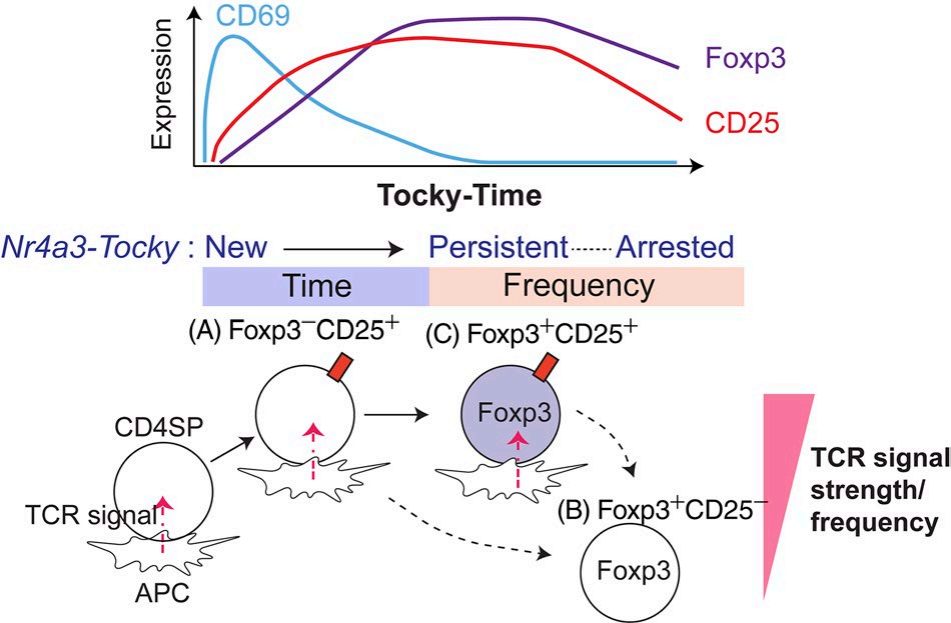
**Model for the influence of TCR signaling dynamics on thymic Treg cell differentiation.**

Collectively, these analyses demonstrate that the major pathway for Treg cell differentiation is from CD25^+^Foxp3^−^ Treg cell precursors in which persistent TCR signals induce Foxp3 expression. The CD25^−^Foxp3^+^ subset is enriched with Foxp3^+^ cells that have received relatively weaker and/or less sustained TCR signals. After TCR signaling, cell surface CD69 expression peaks within 4–8 h. CD25 expression is induced in this early phase in the Timer-blue (New) population and steadily accumulates as T cells receive TCR signals. Foxp3 expression is the most delayed and occurs most efficiently after T cells have persistently interacted with antigen (Fig. 7). Importantly, the Tocky system has for the first time directly shown the temporal sequences of Treg cells differentiation processes, revealing that temporally persistent TCR signals induce CD25^+^Foxp3^+^ Treg cell differentiation. Thus, our investigations show that *Nr4a3*-Tocky and Timer locus analysis effectively unravel the temporal dynamics of T cell differentiation after TCR signals.

### Spontaneous and infrequent TCR signals occur in a minority of peripheral self-reactive T cells from *Nr4a3*-Tocky mice

Considering that Treg cell differentiation occurs through the recognition of cognate antigens in the thymus (Picca et al., 2006) and that these cells receive persistent TCR signals (Fig. 6), we hypothesized that *Nr4a3*-Tocky allows to identify by the persistent dynamics of TCR signals antigen-reactive T cells that recognize their cognate antigen during inflammation. First, we analyzed *Nr4a3*-Tocky mice with WT polyclonal T cell repertoire and found that Timer expression spontaneously occurred in a minority of the T cells from *Nr4a3*-Tocky mice and was mainly blue^–^red^+^ (Fig. S3 A). Remarkably, Timer was expressed by a majority of Treg cell and memory-phenotype T cells (66.1 ± 5.1% and 30.8 ± 6.1%, respectively), whereas it was expressed by only a small proportion of naive T cells (5.7 ± 2.5%; Fig. S3 B). Because both Treg cells and memory-phenotype T cells are self-reactive T cells (Ono and Tanaka, 2016), we hypothesized that all these Timer-positive cells are in fact self-reactive T cells that spontaneously recognize self-antigens and receive TCR signals in the periphery. Interestingly, the Timer Angle of most of Timer-positive cells was 90° (the Arrested locus) irrespective of which T cell fraction they are from (Fig. S3, C and D). This indicates that all these Timer-positive populations received infrequent TCR signals with short durations in a similar manner. To further confirm this, *Nr4a3*-Tocky T cells were adoptively transferred into congenic MHC class II knockout mice to assess the contribution of spontaneous interactions between TCR and self-antigen/MHC class II to Timer expression (Fig. S3 E). As expected, 9 d after transfer, most of Timer expression was lost within MHC class II knockout mice. Collectively, these indicate that the spontaneous Timer expression in CD4^+^ T cells is induced through the infrequent and not-immunogenic interaction of self-reactive TCRs and self-antigen/MHC, which is currently called “tonic TCR signals” (Klein et al., 2014; Ono and Tanaka, 2016).

### The frequency-domain analysis of *Nr4a3*-Tocky mice identify tissue-infiltrating antigen-reactive T cells as cells receiving persistent TCR signals

**Figure 8.**
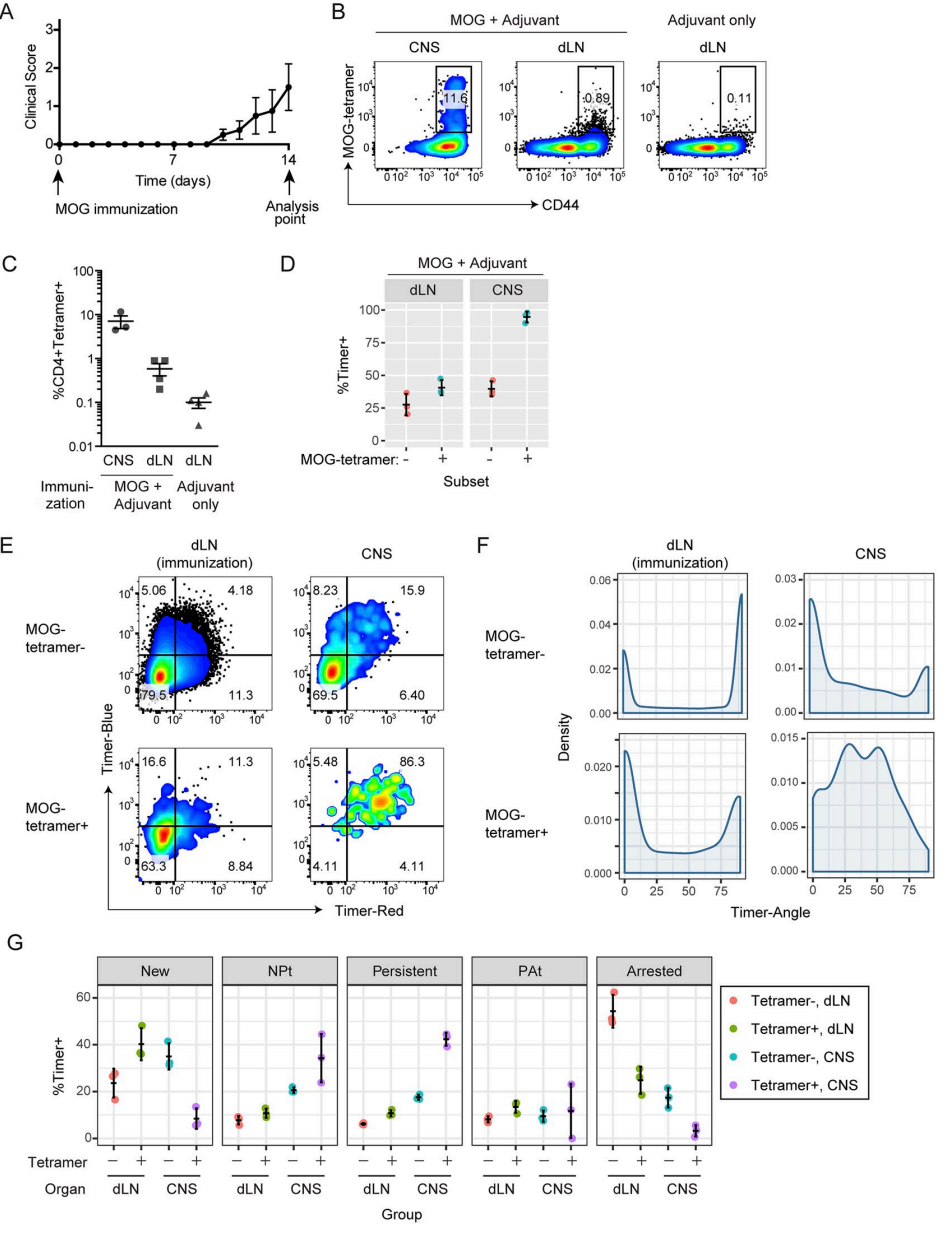
**Tocky mice identify antigen-reactive T cells as cells receiving frequent TCR signals. (A)** Development of clinical symptoms by MOG immunization. **(B)** At day 14, CD4^+^ T cells within the CNS or dLNs were analyzed for MOG-tetramer labeling. Flow cytometry plots depict CD44 versus MOG-tetramer gated on CD4^+^ T cells. **(C)** Percentages of MOG-tetramer–positive cells in dLNs of immunization site and the CNS from MOG-immunized mice or dLNs from control mice with adjuvant only. **(D)** Percentages of Timer-positive cells in MOG-tetramer–positive and –negative cells in either dLNs or CNS from MOG-immunized mice. **(E–G)** MOG-tetramer–positive and –negative cells in either dLNs or CNS from MOG-immunized mice were analyzed for blue and red Timer raw fluorescence by flow cytometry (E) or Timer Angle by density plot (F). **(G)** Timer locus analysis of data from E. *n* = 3 mice; error bars represent mean ± SD. Data are representative of two independent experiments.

Next, we asked whether immunogenic T cell responses have distinct dynamics of TCR signals compared with the infrequent TCR signals in self-reactive T cells. We used a murine model of multiple sclerosis, experimental autoimmune encephalomyelitis, which produces autoimmune T cell responses to myelin basic proteins in the central nervous system (CNS; O’Neill et al., 2006). Upon immunization of myelin oligodendrocyte glycoprotein (MOG), mice developed paralysis within 2 wk, when T cells in the draining lymph nodes (dLNs) of the immunized site and the spinal cord (CNS) were isolated and analyzed (Fig. 8 A). MHC class II tetramer for MOG-specific T cells (designated as MOG-tetramer) stained >10% of CNS-infiltrating T cells, whereas it stained only 0.5% of T cells in dLN (Fig. 8, B and C). This indicates that most of cells in dLN cells are not reactive to MOG, whereas CNS-infiltrating T cells are markedly enriched with MOG-specific T cells, which mediate pathological inflammation (Stromnes and Goverman, 2006). Strikingly, almost 100% of CNS-infiltrating MOG-tetramer^+^ cells expressed Timer, whereas 40% of MOG-tetramer^+^ cells were Timer^+^ in dLN (Fig. 8 D). These findings show that a significantly greater proportion of CNS-infiltrating MOG-specific T cells are actively engaged in TCR signals compared with the dLN. Next, we analyzed the Timer expression in these cells. T cells in dLNs, whether MOG-tetramer positive or negative, showed prominent peaks at the New and Arrested loci (Fig. 8, E and F). This suggests that a significant proportion of T cells receive TCR signals at each moment (therefore producing a peak at the New locus), whereas TCR signals in individual T cells are infrequent (hence producing a peak at the Arrested locus; Fig. 1 E). In contrast, CNS-infiltrating MOG-specific T cells were almost exclusively blue^+^red^+^ and had a single peak between 30° and 60°, whereas CNS-infiltrating tetramer T cells had peaks in the New and Arrested loci (Fig. 8, E and F), showing a similar pattern to those of dLN cells (Fig. 8 F). Most of the MOG-specific T cells in the CNS were found in the Persistent and NP-t loci (Fig. 8 G), further confirming that CNS-infiltrating MOG-specific T cells frequently receive TCR signals.

Collectively, the results above indicate that MOG-specific T cells are engaged with their cognate antigens in the CNS, receiving frequent TCR signals. The accumulation of blue^+^red^+^ cells does not occur in lymph nodes, presumably because both MOG-specific T cells and MOG-presenting antigen-presenting cells (APCs) are rare in lymph nodes. Thus, *Nr4a3*-Tocky identifies antigen-reactive T cells by their transcriptional response in the nucleus. The signal dynamics of these antigen-reactive T cells are distinct from those of bystander or nonreactive T cells and are intriguingly similar to those of thymic CD25^+^Foxp3^+^ Treg cells (Fig. 6 E vs. Fig. 8 E), which are engaged with self–antigen-presenting thymic APCs (namely, under agonistic selection; Klein et al., 2014).

### *Foxp3*-Tocky successfully identifies newly generated Treg cells

**Figure 9.**
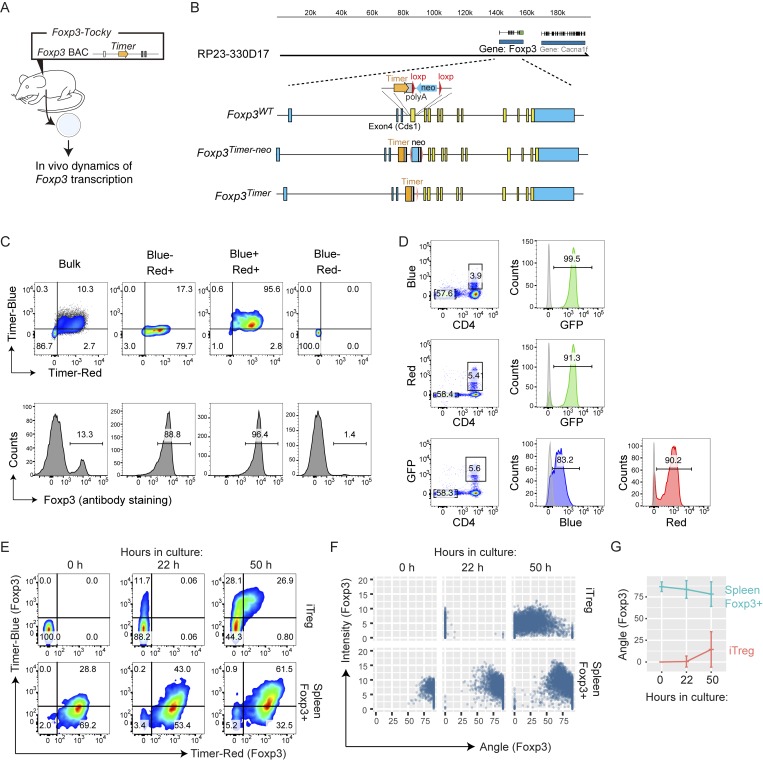
***Foxp3-Tocky* identifies newly generated Treg cells. (A)** Use of *Foxp3-*Tocky mice to investigate in vivo dynamics of *Foxp3* transcription. **(B)** Construct for the generation of Foxp3-Tocky BAC transgenic mice. **(C)** Splenic T cells were sored into blue^−^red+, blue^+^red^+^, and blue^−^red^−^ and analyzed for intracellular Foxp3 proteins. **(D)** *Foxp3-*Tocky mice were crossed with Foxp3-IRES-GFP, and analyzed for the coexpression of GFP and Timer. **(E)** Timer-negative naive T cells and splenic Timer-positive Treg cells from *Foxp3-*Tocky mice were isolated and stimulated by anti-CD3 and anti-CD28 for 0, 22, or 50 h in the presence of IL-2 (and TGF-β for iTreg cells). Flow cytometry plots display raw blue versus red expression during the cultures. **(F and G)** Timer Angle versus Timer Intensity (F) or Timer Angle versus time in the data from E (F).

Next, to address whether the Tocky system can be applied to another gene and to further validate the system, we developed *Foxp3*-Tocky mice using the same approach used for *Nr4a3-*Tocky (Fig. 9, A and B). By investigating Foxp3 protein staining (Fig. 9 C) and *Foxp3^IRES-GFP^ Foxp3-*Tocky double-transgenic mice (Fig. 9 D), Timer expression showed high correlation with GFP and with Foxp3 protein. As expected, when naive CD4^+^ T cells were stimulated in the presence of IL-2 and TGF-β (i.e., induced Treg [iTreg] cell conditions), new Foxp3 expression (blue^+^red^−^) was induced at 22 h, which gained red proteins by 50 h as the Timer chromophore matured (Fig. 9 E). Trigonometric Timer data analysis showed ex vivo splenic Foxp3^+^ T cells from adult mice had high Timer Angle values throughout the culture. In contrast, iTreg cells showed very low Angle values at 22 h, which slowly increased overtime (Fig. 9, F and G). These data indicate that *Foxp3*-Tocky mice can identify the newly differentiating Treg cells that have recently initiated *Foxp3* transcription in vivo, which cannot be achieved through use of existing methods such as GFP reporter mice.

### Foxp3-Tocky reveals that the demethylation of the Foxp3 gene actively occurs when Foxp3 transcription is highly sustained in vivo

**Figure 10.**
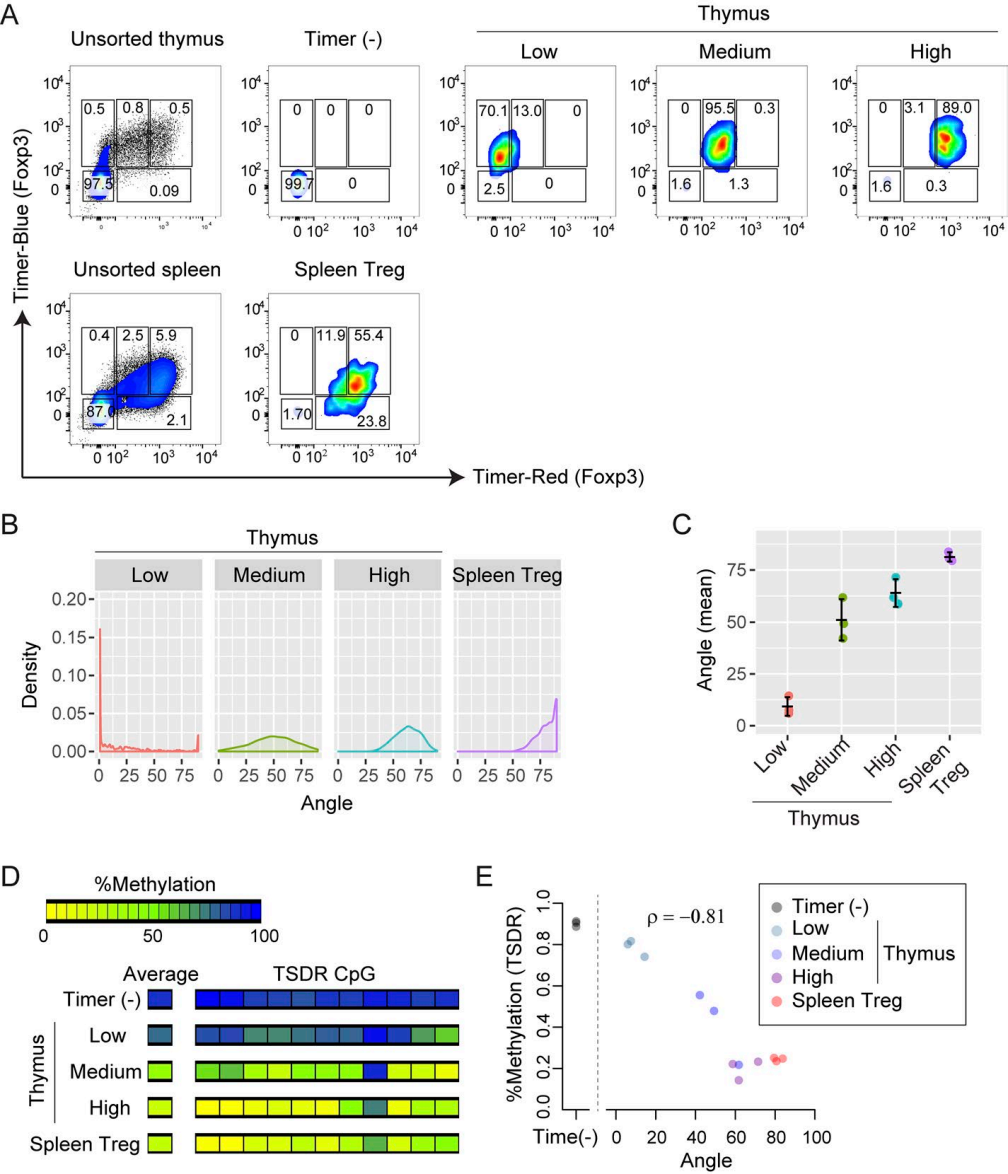
***Foxp3-Tocky*reveals in vivo dynamics of demethylation of the *Foxp3* gene. (A–E)** Thymic cells or splenic Treg cells were sorted according to increasing Tocky time (i.e., Timer Angle) in blue^+^ cells. **(A–C)** Flow cytometry plots of blue versus red fluorescence (A), Timer Angle density plots (B), or mean Timer Angle in the sorted T cell populations (C); *n* = 3. DNA was extracted for methylation analysis, and thymic samples were compared with Timer (−) and splenic Treg cell samples. **(D)** Heatmap showing mean TSDR methylation rates of flow-sorted T cell subsets. **(E)** Mean TSDR methylation rates plotted against mean Timer Angle in sorted T cell subsets (*n* = 3 mice for each subset). Spearman’s correlation coefficient is shown. Data were combined from two experiments.

Next, taking advantage of the Tocky system, we investigated the transcription and methylation of the *Foxp3* gene during Treg cell generation. Although it is known that demethylation can occur within a few hours in cultured cells (Métivier et al., 2008), there is no available method to investigate the in vivo dynamics of demethylation in the mouse body. Immature Treg cells, as identified by high CD24 expression (Marodon and Rocha, 1994), display methylation of the Treg cell–specific demethylated region (TSDR) of the *Foxp3* gene, and loss of CD24 expression is associated with its demethylation (Toker et al., 2013). However, because the in vivo dynamics of CD24 expression is not fully known, it is still unclear how the demethylation of the TSDR dynamically occurs in vivo. Thus, by analyzing the time domain of *Foxp3* transcription using *Foxp3*-Tocky, we investigated the temporal dynamics of the demethylation of the TSDR during thymic Treg cell development. We isolated differentiating thymic T cells by flow-sorting blue^high^ T cells into Timer Angle low, medium, and high (Fig. 10 A). Analysis showed that the isolated populations exhibited distinct Timer Angles from a mean of 10° to 80° (Fig. 10, B and C). DNA was isolated from each population, and their TSDR demethylation was analyzed (Fig. 10 D). Importantly, the newest Foxp3^+^ cells (low) were still mostly methylated and not significantly different from Timer^−^ cells in the degree of TSDR demethylation, whereas rapid demethylation occurred as cells moved to the Persistent locus (Fig. 10 D). Timer Angles showed a strong correlation with TSDR demethylation rates (Spearman’s correlation coefficient ρ = −0.81; Fig. 10 E), indicating that the *Foxp3-*Tocky reporter successfully captures the demethylation dynamics of the *Foxp3* gene. Thus, Foxp3 expression precedes the demethylation of the TSDR region, and the most active demethylation process occurs when *Foxp3* transcription is sustained. Collectively, the *Foxp3-*Tocky reporter successfully identified newly generated Treg cells in vitro and in vivo and also ordered cells from new to relatively aged ones (by the time domain analysis), demonstrating the general applicability of the Tocky system to studies of cellular biology and immunology.

## Discussion

In this study, we have established two uses of the Tocky system. First, the time domain analysis of Tocky allows investigators to determine the relative temporal order of the molecular events after the activation of key signaling pathways and thereby provides a new way to identify immediate precursors and relatively mature cells. Previous studies using fluorescent Timer proteins identified newly generated cells as they expressed solely immature fluorescence without mature fluorescence (Terskikh et al., 2000; Miyatsuka et al., 2011, 2014). In this study, in addition to the rigorous determination of New cells (blue^+^red^−^), we established a quantitative method to measure the relative age of cells that are maturing in vivo. Thus, we determined the temporal sequences of molecular events during thymic Treg cell differentiation after receiving TCR signals using *Nr4a3-*Tocky and identified the major precursor of these cells as the earliest cells among the presumptive precursor populations. In addition, the time domain analysis has allowed us to simultaneously investigate the temporal dynamics of the demethylation process and the transcriptional activities in vivo, identifying and characterizing the earliest stages of Foxp3^+^ Treg cell differentiation. Instead of using a surface marker as an indicator of developmental stage (Toker et al., 2013), we directly investigated the time domain of *Foxp3* transcription and thereby revealed the in vivo dynamics of the demethylation of the *Foxp3* enhancer region: the demethylation process is initiated after *Foxp3* transcription started and it becomes the most active when the transcriptional activity is highly sustained. This means that the Tocky system provides an unprecedented means to investigate the in vivo regulation of molecular mechanisms.

Second, the frequency domain analysis of the Tocky system can identify cells that are undergoing repeated signaling processes. Using *Nr4a3-*Tocky, we showed that thymic T cells that have received sustained TCR signals differentiate into Treg cells. Consistent with these findings, thymic Foxp3^+^ cells show slower and more confined migration by two-photon microscopy compared with other thymic T cells (Le Borgne et al., 2009). These differentiating Foxp3^+^ cells may integrate TCR and other signals from thymic epithelial cells and other APCs. In addition, using *Nr4a3*-Tocky, tissue-infiltrating antigen-specific T cells also accumulate in the Persistent locus, indicating that they frequently interact with cells presenting their cognate antigens. This is also compatible with the findings by intravital microscopic analysis of TCR transgenic T cells that showed that antigen-specific T cells decrease velocity when interacting with antigens (Bousso, 2008). Because the affinity of TCR–ligand interactions determines the dynamics of proximal TCR signaling molecules (Stepanek et al., 2014), future studies should investigate whether and how different TCR affinities are translated into different dynamics of transcription of downstream transcription factors, including Nr4a3. In addition, further studies are required to investigate the temporal dynamics of antigen-specific T cell responses in different contexts such as other autoimmune diseases, allergy, infections, and vaccination. Nevertheless, *Nr4a3*-Tocky enables the investigation of the antigen-specific response of T cells within polyclonal repertoires, revealing in vivo T cell responses at the single-cell level, which may be effective in evaluating the effects of immunotherapy on T cell responses.

In addition, using *Nr4a3*-Tocky, self-reactive T cells are identified as cells that receive infrequent TCR signals in the periphery. The spontaneous TCR signals in self-reactive T cells are historically defined as tonic TCR signals (Stefanová et al., 2002), although their temporal dynamics were unknown. Using *Nr4a3*-Tocky, most of self-reactive T cells are mainly at the Arrested locus, and therefore, the interval of signals is considered to be several times longer than the half-life of blue fluorescence (∼4 h). Further study is required to elucidate the mechanism of the infrequent TCR signals in self-reactive T cells and whether and how TCR and other signals differentiate self-reactive T cells into pathogenic T cells in autoimmune conditions. It is of interest how different frequencies of TCR signals result in the activation of different transcriptional mechanisms.

In general, persistent signals may have distinct biological roles compared with transient signals (Yosef and Regev, 2011), and the Tocky system is an effective tool to investigate these dynamics. For example, sustained TLR4 signaling induces *Il6* transcription effectively, in contrast with transient activities (Litvak et al., 2009); sustained DNA damage, but not transient damage, activates p53 and induces p21 expression and cell cycle arrest (Loewer et al., 2010). To date, such studies used in vitro time-course analyses and/or mathematical modeling to analyze the sustained dynamics of transcription. The Tocky system will benefit studies in cell signaling by providing a means to directly identify and isolate cells receiving persistent signals.

Thus, the Tocky system can be used to dissect the temporal dynamics of cellular differentiation and activation of individual cells by analyzing their time-and frequency domains, providing a measurement of “Tocky time” (Fig. S4), which is in a nonlinear relationship with “real” time, and represents a relative chronological readout for events occurring after a cellular differentiation cue. It is of note that the time and frequency domains of the Tocky time are merged at the Persistent locus, and further mathematical approaches to understand the relationship between these two domains are anticipated. The advantages of the Tocky system over other gene reporters are summarized in Table S1. In the future, Tocky mice for key transcription factors and genes will be promising tools to reveal the in vivo dynamics of gene transcription and cellular differentiation (e.g., Bcl6 for T-follicular helper cells, Rag2 for TCR recombination, and Oct4 for stem cell–ness), which cannot be investigated otherwise. In summary, we have established the Tocky system as a pioneering tool to investigate cellular activation and differentiation and gene dynamics in vivo, which will facilitate studies in cell biology disciplines, including immunology, developmental biology, and stem cell biology.

## Materials and methods

### Transgenesis and mice

The BAC clones RP23-122N18 and RP23-330D17 were obtained from the BACPAC Resources Center at Children’s Hospital Oakland Research Institute and were for generating *Nr4a3-*Tocky and *Foxp3-*Tocky, respectively. BAC DNA was modified by the BAC recombineering approach using the *SW106* strain bacteria (Warming et al., 2005). Two independent lines were established for both of the *Nr4a3-*Tocky and *Foxp3-*Tocky transgenic reporter strains. Both lines exhibited highly similar phenotypes and frequencies of Timer-positive cells.

We used a Timer knock-in knockout approach for BAC transgenic reporter constructs. Precisely, for *Nr4a3-*Tocky, the first coding exon of the *Nr4a3* gene in RP23-122N18 was targeted and replaced with the transgene cassette containing the *Timer (Fast-FT)* gene (Subach et al., 2009), a poly(A) tail, and a floxed neomycin resistance gene (*neo*). For *Foxp3-*Tocky, the first coding exon of the *Foxp3* gene in RP23-330D17 was targeted and replaced by the same transgene cassette. Subsequently, *neo* was excluded by arabinose-inducible Cre expression in SW106 (Warming et al., 2005). BAC DNA was purified by the NucleoBond Xtra Midi kit (Macherey-Nagel) and microinjected into the pronucleus of one-cell embryos from C57BL/6 mice under the approval of the Gene Recombination Experiments Safety Committee of Kyoto University. Founders were screened by genomic PCR, and transgene-positive founders were mated with WT C57BL/6 mice. F1 mice were screened by flow cytometry for Timer expression and subsequently bred to homozygosity with *Foxp3^IRES-GFP^* mice (B6.Cg-*Foxp3^tm1Mal^/J*, 018628; Jackson Laboratory) to generate *Nr4a3-*Tocky:*Foxp3^IRES-GFP^* double-transgenic mice. *OT-II Nr4a3-*Tocky: *Foxp3^IRES-GFP^* mice were similarly generated by crossing *Nr4a3-*Tocky, B6.Cg-*Tg(TcraTcrb)425Cbn*/J, and B6.Cg-*Foxp3^tm1Mal^/J*. MHC class II^ko^/^ko^ (B6.129S2-*H2^dlAb1-Ea^*/J, 003584; The Jackson Laboratory) and congenic CD45.1 (B6.SJL-*Ptprc^a^ Pepc*^b^/BoyJ, 002014; The Jackson Laboratory) were also used. All animal experiments were performed in accordance with local Animal Welfare and Ethical Review Body at Imperial College London and University College London, and all gene recombination experiments were performed under the risk assessment that was approved by the review board at Imperial College London and University College London.

### In vitro Treg cell polarization and mature Treg cell culture

CD4^+^CD44^lo^Foxp3^−^ naive T cells from *Foxp3-*Tocky mice were isolated by cell sorting, and 10^5^ cells were cultured on anti-CD3 (clone 1452C11; 2 µg/ml; eBioscience) and anti-CD28 (clone 37.51; 10 µg/ml; eBioscience)–coated 96-well plates (Corning) in the presence of 100 U/ml rhIL-2 (Roche) and 2 ng/ml rhTGFβ (R&D) for 0–48 h in a final volume of 200 µl RPMI 1640 (Sigma-Aldrich) containing 10% FCS and penicillin/streptomycin (Thermo Fisher Scientific).

Mature Foxp3^+^ Treg cells from *Foxp3-*Tocky mice were isolated by cell sorting, and 10^5^ cells were cultured on anti-CD3 (clone 145.2C11; 2 µg/ml) and anti-CD28 (clone 37.51; 10 µg/ml) –coated 96-well plates in the presence of 100 U/ml rhIL-2 for 0–48 h in a final volume of 200 µl RPMI 1640 containing 10% FCS and penicillin/streptomycin.

### Activation of T and B cells in vitro and the analysis of the half-life of blue-fluorescence

Splenocytes (4 × 10^5^ per well) were cultured on 96-well U-bottom plates coated with 2 µg/ml anti-CD3 and/or 10 µg/ml anti-CD28 for 20 h. Cells were then harvested and replated in the presence of 100 µg/ml cycloheximide (Sigma-Aldrich). At various time points, cells were stained with CD4 and CD8 antibodies, and Timer–blue and Timer–red fluorescence was measured by flow cytometry. In some cultures, 100 U/ml rhIL-2 was added. For polyclonal activation of B cells, 10 µg/ml F(ab′)2 goat anti–mouse IgM (Thermo Fisher Scientific) was added to cultures of splenocytes for 20 h. Cells were then harvested and replated in the presence of 100 µg/ml cycloheximide. At various time points, cells were stained for CD19 and CD3 and CD3^−^CD19^+^ B cells analyzed for Timer–blue and Timer–red fluorescence.

### In vitro T cell activation of OT-II *Nr4a3-*Tocky T cells

CD4^+^ T cells from OT-II *Nr4a3-*Tocky*:Foxp3^GFPKI^* mice were isolated by immunomagnetic cell separation, (StemCell Technologies), and 2 × 10^5^ cells were cultured with 3 × 10^5^ (2:3) CD90.2-depleted splenocytes in the presence of 1, 10, 100, or 1,000 nM Ova_(323–339)_ peptide (Sigma-Aldrich) on 96-well plates (Corning) in a final volume of 200 µl RPMI 1640 containing 10% FCS and penicillin/streptomycin and 55 µM β-mercaptoethanol (Gibco) for the stated time periods. At 24 h, some cells were washed three times and recultured on a fresh plate in the presence of 40 µg/ml anti–MHC class II (clone M5/114; BioXcell) for a further 8–24 h before analysis. The stimulation was terminated in the same manner for periodic stimulation of OT-II Nr4a3.

### Confocal microscopy

Confocal microscopy analysis of stimulated OT-II *Nr4a3*-Tocky T cells was performed using Cytospin as previously described (Ono et al., 2007). In brief, Cytospin slides were prepared with stimulated T cells and fixed by 2% paraformaldehyde for 10 min at room temperature. After washing with PBS, fluorescence images were obtained by the confocal system LSM510 invert (ZEISS) using a Plan Apochromat 20×/0.8 objective. The laser-scanning microscopy image files were converted into TIFF format and further analyzed by FIJI (ImageJ; National Institutes of Health; Schindelin et al., 2012). For quantitative analysis of fluorescence intensities in single cells, individual cells were manually identified as region of interest using light-field differential interference contrast images. It is of note that because GFP^+^Foxp3^+^ cells were very rare in stimulated OT-II *Nr4a3*-Tocky cells, we used GFP images as a dump, excluding cells with high autofluorescence (Fig. S2, arrowheads). Measurement of mean fluorescence intensities were obtained by redirecting them to thresholded grayscale images for Timer–blue and Timer–red fluorescence. The same threshold value was applied to all the images from the same channel. Mean fluorescence intensities were logged to produce histograms and 2D plots. Color images were enhanced for brightness by applying the same linear adjustment to all images from the same channel.

### Experimental autoimmune encephalomyelitis

Experimental autoimmune encephalomyelitis was induced by subcutaneous injection of 200 µg MOG_35–55_ (Sigma-Aldrich) emulsified in Freund’s adjuvant (Sigma-Aldrich) containing 4 mg/ml heat-killed mycobacteria (Invivogen). Control mice received complete Freund’s adjuvant alone, emulsified with sterile water. On days 0 and 2, mice received 200 ng pertussis toxin i.p. (EMD Millipore). Mice were monitored for the development of clinical symptoms (0, no clinical symptoms; 0.5, tip of the tail is limp; 1, limp tail; 1.5, limp tail and hindleg inhibition; 2, limp tail and weakness in hindlegs; 2.5, limp tail and dragging of hind legs; 3, limp tail and paralysis of hindlegs). The severity limit of the protocol was 3.

For isolating cells from the spinal cord, mice were culled and the left ventricle perfused with ice-cold PBS. Spinal cords were removed and forced through a 70-µm cell strainer. CNS lymphocytes were separated from myelin using a 30%/70% Percoll gradient (Sigma-Aldrich). Mononuclear cells were removed from the interphase and washed and resuspended in 10% RPMI for labeling.

### Tetramer staining

dLN or CNS mononuclear cells isolated by Percoll gradient were incubated at 37°C for 15 min in the presence of 50 nM dasatinib (Lissina et al., 2009). 1 in 100 dilution of APC-labeled mouse I-A^b^ MOG peptide 38–49 (GWYRSPFSRVVH, I-Ab MOG_38–49_; National Institutes of Health Tetramer Core) was added to the cells and further incubated for 30 min at 37°C. Cells were then washed and stained for viability dye and surface markers on ice.

### Flow cytometric analysis and cell sorting

After spleen or thymus removal, organs were forced through a 70-µm cell strainer to generate a single-cell suspension. For splenocyte preparations, an RBC-lysis stage was used. Staining was performed on a V-bottom 96-well plate or in 15-ml falcon tubes for cell sorting. Analysis was performed on a BD Fortessa III instrument. The blue form of the Timer protein was detected in the blue (450/40 nm) channel excited off the 405-nm laser. The red form of Timer protein was detected in the mCherry (610/20) channel excited off the 561-nm laser. For all experiments, a fixable eFluor 780 fluorescent viability dye was used (eBioscience). The following directly conjugated antibodies were used in these experiments: CD3 PerCPcy5.5 (clone 145.2C11; TONBO) CD4 APC (clone RM4-5; eBioscience), CD4 Alexa Fluor 700 and CD4 BUV395 (clone GK1.5; BD; clone RM4-5; BioLegend), CD8 PE-Cy7 or APC (clone 53–6.7; BioLegend), CD8 BUV737 (clone 53–6.7; BD) TCRβ FITC and Alexa Fluor 700 (clone H57-597; BioLegend), TCRβ BUV737 (clone H57-597; BD), CD19 APC (clone 6D5; BioLegend), CD25 PerCPcy5.5 (PC61.5; eBioscience) or PE-Cy7 (PC61.5; Tombo Bioscience), CD44 APC (clone IM7; eBioscience) or Alexa Fluor 700 (clone IM7; BioLegend), CD69 APC (H1.2F3; eBioscience) and Foxp3 APC (clone FJK-16s; eBioscience), CD45.1 PE-Cy7 (clone A20; BioLegend), CD45.2 APC (clone 104; BioLegend), and CD45RB PE-Cy7 (clone C363-16A). For *Foxp3-*Tocky validation, RBC-lysed splenocytes were stained with eFluor 780 viability dye before staining for APC-conjugated CD4 and FITC-conjugated TCRβ. Live CD4^+^TCRβ^+^ cells were gated and then sorted into four fractions: Timer(blue^+^red^−^), Timer (blue^+^red^+^), Timer(blue^−^red^+^), and Timer(blue^−^red^−^). Cells were then fixed and labeled with APC-conjugated Foxp3 using the eBioscience Foxp3 fixation and permeabilization kit according to the manufacturer’s instructions. In some experiments, cells were labeled with an eFluor 670 proliferation dye (eBioscience) at a concentration of 5 µM for 5 min at room temperature.

### Adoptive transfer experiments

CD4^+^CD25^−^CD44^lo^Foxp3^−^T cells from OT-II *Nr4a3*-Tocky mice were isolated by cell sorting on FACS Aria III, and between 1 and 5 M cells were adoptively transferred i.p. to congenic CD45.1 recipients, which were fed Ova (Sigma-Aldrich) at a concentration of 1% in water. For MHC class II knockout experiments, CD4^+^ T cells were isolated by immunomagnetic selection (Stem Cell Technologies) and 3–5 M cells injected i.p. into congenic (CD45.1) MHC class II^ko^/^ko^ mice. MHC class II^ko^/^wt^ mice were used as controls. Adoptively transferred T cells were defined as CD45.1^−^CD45.2^+^CD4^+^TCRβ^+^ cells.

### DNA methylation analysis

DNA was extracted from sorted samples using QIAGEN DNeasy kit according to the manufacturer’s instructions. 100 ng DNA was bisulfite treated using the Epitect Bilsulfite kit (QIAGEN) and used as a template for amplification of the TSDR. TSDR was amplified using the primers Foxp3 TSDR forward, 5′-ATTTGAATTGGATATGGTTTGT-3′; and reverse, 5′-AACCTTAAACCCCTCTAACATC-3′ (Floess et al., 2007) and the following cycling conditions: 94°C for 1 min followed by 40 cycles of 94°C for 15 s, 54°C for 30 s, and 68°C 30 s and a final extension phase of 68°C for 15 min. PCR amplicons were purified and then underwent Sanger sequencing using the reverse primer by Source BioScience. Sequence traces and CpG methylation rates were analyzed and determined by ESME software (Epigenomics; Lewin et al., 2004).

### CCA

The gene signature and sample scores in Fig. S1 were calculated by cross-dataset analysis using CCA (Ono et al., 2014). In brief, the expression data of GSE15907 (Painter et al., 2011) was regressed onto the log2 fold change of activated CD4^+^ T cells (2 h after activation) and naive T cells from GSE48210 (Li et al., 2013) as the explanatory variable, correspondence analysis was performed for the regressed data, and correlation analysis was done between the new axis and the explanatory variable. CCA was performed by the CRAN package *vegan* as previously described (Ono et al., 2014). The analysis was undertaken using only transcription factor genes, which were selected by the Gene Ontology database by including the genes that are tagged with GO: 0003677 (DNA binding) and GO: 0005634 (nucleus) and not with GO: 0016020 (membrane) using the Bioconductor package *GO.db*.

### Timer data analysis

#### Overview of Timer data analysis

Timer data analysis is composed of the following three steps: (1) data preprocessing and scaling/normalization of blue and red fluorescence data; (2) trigonometric data transformation of blue and red fluorescence, which transform data into Timer Angle and Timer Intensity using the polar coordinate; and (3) data export, statistical analysis and visualization. Timer data analysis will be performed by importing those csv files into R (R Core Team 2016). A series of data preprocessing, normalization and transformation results in Timer normalized data, which are further analyzed for statistics, visualization, and quality control processes. Finally, the code exports these quality control and statistical results, the cell number data of total cells and Timer-positive cells in each file, and a matrix containing Timer Angle and Timer Intensity data and all the fluorescence data for individual cells in each file (Fig. S5 A).

#### Data import

Flow cytometric data are gated for T cell populations (e.g., CD4^+^ T cells) by an external program such as FlowJo or the Bioconductor package *flowCore* and batch exported as csv files, including a negative control file and a forward scatter (FSC) control file (see below). The R codes import all files and produce and work on the dataset as an R object.

#### Data preprocessing

Data preprocessing for Timer data analysis is composed of the following three steps: (1) FSC correction, (2) data thresholding, and (3) data normalization.

##### FSC correction

Our investigations showed that blue autofluorescence increases as FSC increases, which indicates that larger cells have higher blue signals (Fig. S5 B). This effect was more remarkable in blue than red, which can result in underestimation of Timer Angle (e.g., in activated T cells, which have larger cell sizes). Accordingly, the code incorporates the function to perform FSC correction by applying a linear regression to FSC (*x*) and either blue (*b*) or red (*r*):

$$b=\;\alpha_{B}+\;\beta_{B}x$$

or

$$r=\alpha_{R}+\;\beta_{R}x.$$

Thus, FSC-corrected blue (*b_c_*) and FSC-corrected red (*r_c_*) are obtained by

$$b_{c}=b-\beta_{B}x$$

and

$$r_{c}=r-\beta_{R}x.$$

##### Data thresholding

The instrument setting of flow cytometry is determined by manually adjusting the amplification parameters so that the autofluorescence signals of negative cells have positive values and the threshold value for positive signals can be visually determined. In the standard practice of flow cytometric analysis, the threshold value for each fluorescence is determined using a negative control cell sample, so that a certain small proportion of cells are identified as positive (e.g., 0.5% of the parent population; Fujii et al., 2016). These negative signals are problematic for trigonometric data transformation because blue^low^red^−^ and blue^−^red^low^ cells can have various Timer Angle values, which is biologically meaningless (Fig. S5 C). Accordingly, the negative signals of FSC-corrected blue (*b_c_*) and FSC-corrected red data (*r_c_*) will be collapsed using the threshold value *n_B_* and *n_R_*, respectively:

$$b_{t}=\{\begin{array}{c} b_{c}-n_{B},\;\;if\;b_{c}>n_{B}\\ 0,\;\;if\;b_{c}\leq n_{B} \end{array}$$

and

$$r_{t}=\{\begin{array}{c} r_{c}-n_{R},\;\;if\;r_{c}>n_{R}\\ 0,\;\;if\;r_{c}\leq n_{R} \end{array}.$$

In Fig. S5 C, Timer Angle calculation with or without data thresholding is compared on uniformly distributed cell data. In this figure, it is assumed that each cell has a discrete integer value between 0 and 100 for blue and another value for red (i.e., there are 10,201 cells in the blue–red plane) and that the barycenter of negative control cells is (10, 10). When data are processed by blue–red normalization without applying data thresholding, cells can have various Timer Angle values around the barycenter of negative control cells, and thus, cells in the negative quadrant gate (the lower left quadrant of pink lines) can have all the different Timer Angle values (Fig. S5 C, top left) and have all the Timer loci (Fig. S5 C, top right). These variations in blue^−^ red^−^ cells are biologically meaningless. In Fig. S5 C, bottom left, data were first thresholded (at blue = 18 and red = 18 in this example), and thereafter blue and red data were normalized. The thresholding removes all the blue^−^ red^−^ cells. All the blue^−^ red^+^ cells have Timer Angle value 90, whereas all the blue^+^ red^−^ have Timer Angle value 0.

##### Log transformation

After thresholding negative signals, Timer fluorescence data are log transformed by the function *log*(*x* + 1).

##### Blue–red normalization

The autofluorescence data of blue and red fluorescence in negative control sample are used to estimate the deviation of blue and red signals in each dataset. Assuming that the SD of blue and red fluorescence in negative control is σ_B_ and σ_R_, normalized blue and red data *b_n_* and *r_n_* are obtained as follows:

$$b_{n}=\frac{b_{t}}{\sigma_{B}}$$

or

$$r_{n}=\frac{r_{t}}{\sigma_{R}}.$$

#### Trigonometric data transformation

Lastly, using a trigonometric function, normalized blue and red data will be transformed into Timer Angle and Timer Intensity data. Timer Angle θ is defined as the angle from the normalized blue axis and has the value between 0° and 90°, or [0, $\frac{\pi }{2}$ ]. Timer Intensity δ is the distance from the origin of normalized blue and normalized red axes (Fig. S5 D). By definition, cells in the origin (0, 0) are negative for Timer protein and removed from further analysis. Thus, the R code automatically identifies Timer-positive cells:

$$\delta =\;\sqrt{b_{n}{}^{2}+r_{n}{}^{2}}$$

and

$$\theta =\cos^{-1}(\frac{b_{n}}{\delta }).$$

#### Timer locus analysis

Timer locus analysis allows the translation of Timer data into transcriptional dynamics and the biological interpretation of individual cells (Fig. S5 E). The five Timer loci are designated as follows: New = 0°, NP-t = (0°, 30°), Persistent [30°, 60°], PA-t [60°, 90°], and Arrested = 90° (Fig. S5 F). Nonparametric tests for Timer locus were performed by the *CRAN* package. A Kruskal test was applied to data with more than two experimental groups, and subsequently, post hoc Dunn’s test was applied.

### Computer simulation of Timer fluorescence

Computer simulation of GFP and Timer expression was performed by time course analysis of a series of first-order equations involving the function for transcript (mRNA) *X* and translated Timer proteins including the colorless-form *C*, the blue form *B*, the intermediate form *I* (which does not emit fluorescence), and the red form *R*, or translated GFP protein *G* and the decayed protein *D*. Thus, a liner kinetic model for Timer protein was constructed as previously reported (Subach et al., 2009):

$$X\overset{k_{C}}{\to }C\overset{k_{B}}{\to }B\overset{k_{I}}{\to }I\overset{k_{R}}{\to }R\overset{k_{D}}{\to }D,$$

and for GFP:

$$X\overset{k_{G}}{\to }G\overset{k_{D}}{\to }D.$$

The amounts of blue- and red-form proteins are

$$\frac{dB}{dt}=\;k_{B}C-\;k_{I}B$$

and

$$\frac{dR}{dt}=\;k_{R}I-\;k_{D}R.$$

Similarly, the amounts of other intermediate form proteins were modeled in a similar manner, and time-course analysis was performed by the CRAN package *deSolve*. Analytically, the amounts of blue- and red-form proteins are as follows:

$$B\left(t\right)=e^{-k_{I}t\;}\int \text{​}e^{k_{I}s\;}k_{B}C\left(s\right)ds+k_{B}e^{-k_{I}t\;}$$

and

$$R\left(t\right)=e^{-k_{D}t\;}\int \text{​}e^{k_{D}s\;}k_{R}I\left(s\right)ds+k_{R}e^{-k_{D}t}.$$

Thus, blue- and red-form proteins are dependent on the supply from their precursor proteins, and when transcription is terminated and no precursor protein is supplied, they are expected to exponentially decay at the rate *k_B_* and *k_R_*. For Fig. 1 C, the parameters from the preceding study (Subach et al., 2009) were used to perform time-course analysis. For GFP, the translation rate was set to be the same with Timer, *k_G_* = 3.0 × 10^−1^/*h*, whereas the decay rate was set to be *k_E_* = 1.3 × 10^−2^/*h* as previously reported (Sacchetti et al., 2001). Density plots for the distribution of Timer Angle (Fig. 1 G) were generated by testing different frequencies and intermittent expressions, assuming that signals are conveyed to cells for 7 d by the temporal dynamics shown in each figure and that each cells is at a random time point in this duration.

### Statistical analysis and data visualization

Statistical analysis was performed on R or Prism 6 (GraphPad Software). Percentage data for Timer-positive and Timer locus analysis was analyzed by Mann-Whitney *U* test or Kruskal-Wallis test with Dunn’s multiple comparisons using the CRAN package *PMCMR*. Samples with <18 Timer-positive cells were not included in the analysis. Student’s *t* test was used for comparison of two means. For comparison of more than two means, a one-way ANOVA with Tukey’s post hoc test was applied using the CRAN package *Stats*. Scatterplots were produced by the CRAN packages *ggplot2* and *graphics*. Dose–response data in Fig. 3 G were fitted to a dose–response curve using the three-parameter log-logistic function of the CRAN package *drc* as previously described (Yoshioka et al., 2012). All computations were performed on a Mac (version 10.11.6). Adobe Illustrator (CS5) was used for compiling figures and designing schematic figures. Variance is reported as SD or SEM unless otherwise stated.

### Online supplemental material

Fig. S1 shows that CCA identifies *Nr4a3* as a downstream target of TCR signaling. Fig. S2 shows that confocal microscopy analysis of OT-II *Nr4a3*-Tocky T cells stimulated at various frequencies. Fig. S3 shows that steady-state TCR signaling is restricted to memory-like T cells in vivo and dependent on MHC class II. Fig. S4 shows a summary of Tocky technology. Fig. S5 shows Timer data analysis methods. Table S1 lists differences between Tocky, fate-mapper, and GFP/FP reporters.

## Supplementary Material

Supplemental Materials (PDF)

## References

[bib1] Boomer J.S., and Green J.M. 2010 An enigmatic tail of CD28 signaling. Cold Spring Harb. Perspect. Biol. 2:a002436 10.1101/cshperspect.a002436 20534709 PMC2908766

[bib2] Bousso P. 2008 T-cell activation by dendritic cells in the lymph node: lessons from the movies. Nat. Rev. Immunol. 8:675–684. 10.1038/nri2379 19172690

[bib3] Burchill M.A., Yang J., Vogtenhuber C., Blazar B.R., and Farrar M.A. 2007 IL-2 receptor beta-dependent STAT5 activation is required for the development of Foxp3+ regulatory T cells. J. Immunol. 178:280–290. 10.4049/jimmunol.178.1.280 17182565

[bib4] Cantrell D. 2015 Signaling in lymphocyte activation. Cold Spring Harb. Perspect. Biol. 7:7 10.1101/cshperspect.a018788 PMC4448613 26032717

[bib5] Donà E., Barry J.D., Valentin G., Quirin C., Khmelinskii A., Kunze A., Durdu S., Newton L.R., Fernandez-Minan A., Huber W., 2013 Directional tissue migration through a self-generated chemokine gradient. Nature. 503:285–289. 10.1038/nature12635 24067609

[bib6] Floess S., Freyer J., Siewert C., Baron U., Olek S., Polansky J., Schlawe K., Chang H.D., Bopp T., Schmitt E., 2007 Epigenetic control of the foxp3 locus in regulatory T cells. PLoS Biol. 5:e38 10.1371/journal.pbio.0050038 17298177 PMC1783672

[bib7] Fujii H., Josse J., Tanioka M., Miyachi Y., Husson F., and Ono M. 2016 Regulatory T Cells in Melanoma Revisited by a Computational Clustering of FOXP3+ T Cell Subpopulations. J. Immunol. 196:2885–2892. 10.4049/jimmunol.1402695 26864030 PMC4777917

[bib8] Hoppe P.S., Coutu D.L., and Schroeder T. 2014 Single-cell technologies sharpen up mammalian stem cell research. Nat. Cell Biol. 16:919–927. 10.1038/ncb3042 25271480

[bib9] Hoyer K.K., Dooms H., Barron L., and Abbas A.K. 2008 Interleukin-2 in the development and control of inflammatory disease. Immunol. Rev. 226:19–28. 10.1111/j.1600-065X.2008.00697.x 19161413

[bib10] Hsieh C.S., Lee H.M., and Lio C.W. 2012 Selection of regulatory T cells in the thymus. Nat. Rev. Immunol. 12:157–167. 10.1038/nri3155 22322317

[bib11] Khmelinskii A., Keller P.J., Bartosik A., Meurer M., Barry J.D., Mardin B.R., Kaufmann A., Trautmann S., Wachsmuth M., Pereira G., 2012 Tandem fluorescent protein timers for in vivo analysis of protein dynamics. Nat. Biotechnol. 30:708–714. 10.1038/nbt.2281 22729030

[bib12] Klein L., Kyewski B., Allen P.M., and Hogquist K.A. 2014 Positive and negative selection of the T cell repertoire: what thymocytes see (and don’t see). Nat. Rev. Immunol. 14:377–391. 10.1038/nri3667 24830344 PMC4757912

[bib13] Koechlein C.S., Harris J.R., Lee T.K., Weeks J., Fox R.G., Zimdahl B., Ito T., Blevins A., Jung S.H., Chute J.P., 2016 High-resolution imaging and computational analysis of haematopoietic cell dynamics in vivo. Nat. Commun. 7:12169 10.1038/ncomms12169 27425143 PMC4960315

[bib14] Kohwi M., and Doe C.Q. 2013 Temporal fate specification and neural progenitor competence during development. Nat. Rev. Neurosci. 14:823–838. 10.1038/nrn3618 24400340 PMC3951856

[bib15] Krummel M.F., Bartumeus F., and Gérard A. 2016 T cell migration, search strategies and mechanisms. Nat. Rev. Immunol. 16:193–201. 10.1038/nri.2015.16 26852928 PMC4869523

[bib16] Kurd N., and Robey E.A. 2016 T-cell selection in the thymus: a spatial and temporal perspective. Immunol. Rev. 271:114–126. 10.1111/imr.12398 27088910 PMC4938245

[bib17] Le Borgne M., Ladi E., Dzhagalov I., Herzmark P., Liao Y.F., Chakraborty A.K., and Robey E.A. 2009 The impact of negative selection on thymocyte migration in the medulla. Nat. Immunol. 10:823–830. 10.1038/ni.1761 19543275 PMC2793676

[bib18] Lewin J., Schmitt A.O., Adorján P., Hildmann T., and Piepenbrock C. 2004 Quantitative DNA methylation analysis based on four-dye trace data from direct sequencing of PCR amplificates. Bioinformatics. 20:3005–3012. 10.1093/bioinformatics/bth346 15247106

[bib19] Li L., Nishio J., van Maurik A., Mathis D., and Benoist C. 2013 Differential response of regulatory and conventional CD4^+^ lymphocytes to CD3 engagement: clues to a possible mechanism of anti-CD3 action? J. Immunol. 191:3694–3704. 10.4049/jimmunol.1300408 23986534 PMC3932531

[bib20] Lio C.-W.J., and Hsieh C.-S. 2008 A two-step process for thymic regulatory T cell development. Immunity. 28:100–111. 10.1016/j.immuni.2007.11.021 18199417 PMC2248212

[bib21] Lissina A., Ladell K., Skowera A., Clement M., Edwards E., Seggewiss R., van den Berg H.A., Gostick E., Gallagher K., Jones E., 2009 Protein kinase inhibitors substantially improve the physical detection of T-cells with peptide-MHC tetramers. J. Immunol. Methods. 340:11–24. 10.1016/j.jim.2008.09.014 18929568 PMC3052435

[bib22] Litvak V., Ramsey S.A., Rust A.G., Zak D.E., Kennedy K.A., Lampano A.E., Nykter M., Shmulevich I., and Aderem A. 2009 Function of C/EBPdelta in a regulatory circuit that discriminates between transient and persistent TLR4-induced signals. Nat. Immunol. 10:437–443. 10.1038/ni.1721 19270711 PMC2780024

[bib23] Loewer A., Batchelor E., Gaglia G., and Lahav G. 2010 Basal dynamics of p53 reveal transcriptionally attenuated pulses in cycling cells. Cell. 142:89–100. 10.1016/j.cell.2010.05.031 20598361 PMC3003696

[bib24] Marodon G., and Rocha B. 1994 Generation of mature T cell populations in the thymus: CD4 or CD8 down-regulation occurs at different stages of thymocyte differentiation. Eur. J. Immunol. 24:196–204. 10.1002/eji.1830240131 7912676

[bib25] Métivier R., Gallais R., Tiffoche C., Le Péron C., Jurkowska R.Z., Carmouche R.P., Ibberson D., Barath P., Demay F., Reid G., 2008 Cyclical DNA methylation of a transcriptionally active promoter. Nature. 452:45–50. 10.1038/nature06544 18322525

[bib26] Miyatsuka T., Kosaka Y., Kim H., and German M.S. 2011 Neurogenin3 inhibits proliferation in endocrine progenitors by inducing Cdkn1a. Proc. Natl. Acad. Sci. USA. 108:185–190. 10.1073/pnas.1004842108 21173230 PMC3017196

[bib27] Miyatsuka T., Matsuoka T.A., Sasaki S., Kubo F., Shimomura I., Watada H., German M.S., and Hara M. 2014 Chronological analysis with fluorescent timer reveals unique features of newly generated β-cells. Diabetes. 63:3388–3393. 10.2337/db13-1312 24834978 PMC4392905

[bib28] Moran A.E., Holzapfel K.L., Xing Y., Cunningham N.R., Maltzman J.S., Punt J., and Hogquist K.A. 2011 T cell receptor signal strength in Treg and iNKT cell development demonstrated by a novel fluorescent reporter mouse. J. Exp. Med. 208:1279–1289. 10.1084/jem.20110308 21606508 PMC3173240

[bib29] O’Neill E.J., Day M.J., and Wraith D.C. 2006 IL-10 is essential for disease protection following intranasal peptide administration in the C57BL/6 model of EAE. J. Neuroimmunol. 178:1–8. 10.1016/j.jneuroim.2006.05.030 16872684 PMC3399771

[bib30] Oh H., and Ghosh S. 2013 NF-κB: roles and regulation in different CD4(+) T-cell subsets. Immunol. Rev. 252:41–51. 10.1111/imr.12033 23405894 PMC3576882

[bib31] Ono M., and Tanaka R.J. 2016 Controversies concerning thymus-derived regulatory T cells: fundamental issues and a new perspective. Immunol. Cell Biol. 94:3–10. 10.1038/icb.2015.65 26215792 PMC4650266

[bib32] Ono M., Yaguchi H., Ohkura N., Kitabayashi I., Nagamura Y., Nomura T., Miyachi Y., Tsukada T., and Sakaguchi S. 2007 Foxp3 controls regulatory T-cell function by interacting with AML1/Runx1. Nature. 446:685–689. 10.1038/nature05673 17377532

[bib33] Ono M., Tanaka R.J., and Kano M. 2014 Visualisation of the T cell differentiation programme by Canonical Correspondence Analysis of transcriptomes. BMC Genomics. 15:1028 10.1186/1471-2164-15-1028 25428805 PMC4258272

[bib34] Painter M.W., Davis S., Hardy R.R., Mathis D., and Benoist C.. Immunological Genome Project Consortium 2011 Transcriptomes of the B and T lineages compared by multiplatform microarray profiling. J. Immunol. 186:3047–3057. 10.4049/jimmunol.1002695 21307297 PMC3140206

[bib35] Picca C.C., Larkin J. III, Boesteanu A., Lerman M.A., Rankin A.L., and Caton A.J. 2006 Role of TCR specificity in CD4+ CD25+ regulatory T-cell selection. Immunol. Rev. 212:74–85. 10.1111/j.0105-2896.2006.00416.x 16903907

[bib36] Roncagalli R., Hauri S., Fiore F., Liang Y., Chen Z., Sansoni A., Kanduri K., Joly R., Malzac A., Lähdesmäki H., 2014 Quantitative proteomics analysis of signalosome dynamics in primary T cells identifies the surface receptor CD6 as a Lat adaptor-independent TCR signaling hub. Nat. Immunol. 15:384–392. 10.1038/ni.2843 24584089 PMC4037560

[bib37] Sacchetti A., El Sewedy T., Nasr A.F., and Alberti S. 2001 Efficient GFP mutations profoundly affect mRNA transcription and translation rates. FEBS Lett. 492:151–155. 10.1016/S0014-5793(01)02246-3 11248254

[bib38] Schindelin J., Arganda-Carreras I., Frise E., Kaynig V., Longair M., Pietzsch T., Preibisch S., Rueden C., Saalfeld S., Schmid B., 2012 Fiji: an open-source platform for biological-image analysis. Nat. Methods. 9:676–682. 10.1038/nmeth.2019 22743772 PMC3855844

[bib39] Stefanová I., Dorfman J.R., and Germain R.N. 2002 Self-recognition promotes the foreign antigen sensitivity of naive T lymphocytes. Nature. 420:429–434. 10.1038/nature01146 12459785

[bib40] Stepanek O., Prabhakar A.S., Osswald C., King C.G., Bulek A., Naeher D., Beaufils-Hugot M., Abanto M.L., Galati V., Hausmann B., 2014 Coreceptor scanning by the T cell receptor provides a mechanism for T cell tolerance. Cell. 159:333–345. 10.1016/j.cell.2014.08.042 25284152 PMC4304671

[bib41] Stromnes I.M., and Goverman J.M. 2006 Passive induction of experimental allergic encephalomyelitis. Nat. Protoc. 1:1952–1960. 10.1038/nprot.2006.284 17487182

[bib42] Subach F.V., Subach O.M., Gundorov I.S., Morozova K.S., Piatkevich K.D., Cuervo A.M., and Verkhusha V.V. 2009 Monomeric fluorescent timers that change color from blue to red report on cellular trafficking. Nat. Chem. Biol. 5:118–126. 10.1038/nchembio.138 19136976 PMC2662996

[bib43] Tai X., Erman B., Alag A., Mu J., Kimura M., Katz G., Guinter T., McCaughtry T., Etzensperger R., Feigenbaum L., 2013 Foxp3 transcription factor is proapoptotic and lethal to developing regulatory T cells unless counterbalanced by cytokine survival signals. Immunity. 38:1116–1128. 10.1016/j.immuni.2013.02.022 23746651 PMC3700677

[bib44] Terskikh A., Fradkov A., Ermakova G., Zaraisky A., Tan P., Kajava A.V., Zhao X., Lukyanov S., Matz M., Kim S., 2000 “Fluorescent timer”: protein that changes color with time. Science. 290:1585–1588. 10.1126/science.290.5496.1585 11090358

[bib45] Toker A., Engelbert D., Garg G., Polansky J.K., Floess S., Miyao T., Baron U., Düber S., Geffers R., Giehr P., 2013 Active demethylation of the Foxp3 locus leads to the generation of stable regulatory T cells within the thymus. J. Immunol. 190:3180–3188. 10.4049/jimmunol.1203473 23420886

[bib46] Trapnell C., Cacchiarelli D., Grimsby J., Pokharel P., Li S., Morse M., Lennon N.J., Livak K.J., Mikkelsen T.S., and Rinn J.L. 2014 The dynamics and regulators of cell fate decisions are revealed by pseudotemporal ordering of single cells. Nat. Biotechnol. 32:381–386. 10.1038/nbt.2859 24658644 PMC4122333

[bib47] Walker L.S.K., and Sansom D.M. 2011 The emerging role of CTLA4 as a cell-extrinsic regulator of T cell responses. Nat. Rev. Immunol. 11:852–863. 10.1038/nri3108 22116087

[bib48] Warming S., Costantino N., Court D.L., Jenkins N.A., and Copeland N.G. 2005 Simple and highly efficient BAC recombineering using galK selection. Nucleic Acids Res. 33:e36 10.1093/nar/gni035 15731329 PMC549575

[bib49] Weissler K.A., and Caton A.J. 2014 The role of T-cell receptor recognition of peptide:MHC complexes in the formation and activity of Foxp3^+^ regulatory T cells. Immunol. Rev. 259:11–22. 10.1111/imr.12177 24712456 PMC4034456

[bib50] Yosef N., and Regev A. 2011 Impulse control: temporal dynamics in gene transcription. Cell. 144:886–896. 10.1016/j.cell.2011.02.015 21414481 PMC3148525

[bib51] Yoshioka Y., Ono M., Osaki M., Konishi I., and Sakaguchi S. 2012 Differential effects of inhibition of bone morphogenic protein (BMP) signalling on T-cell activation and differentiation. Eur. J. Immunol. 42:749–759. 10.1002/eji.201141702 22144105

