# Supplementary material for: A timer for analyzing temporally dynamic changes in transcription during differentiation in vivo
Source: J Cell Biol. 2018 Aug 6;217(8):2931–50. doi: 10.1083/jcb.201711048 (PMC6080944; doi:10.1083/jcb.201711048)
Supplement: Supplemental Materials (PDF) [file JCB_201711048_sm.pdf]

Supplemental material

Bending et al., https://doi.org/10.1083/jcb.201711048

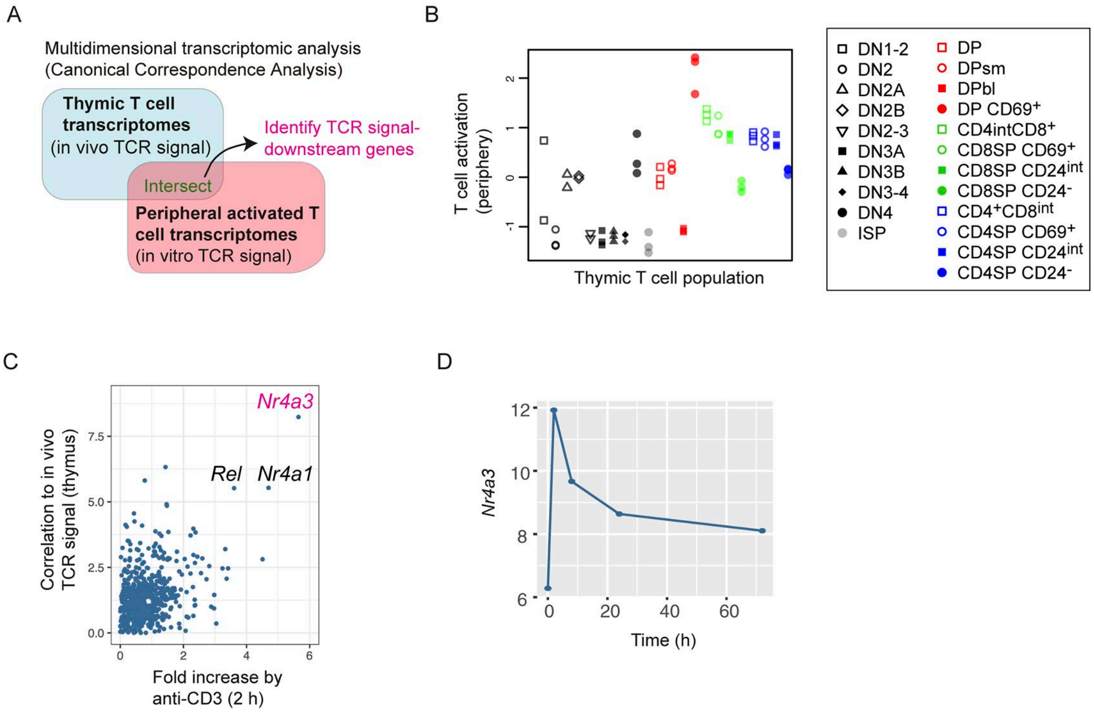

Figure S1. **CCA identifies *Nr4a3* as a downstream target of TCR signaling. (A)** Schematic showing rationale for identifying genes that are downstream of TCR signaling. Cross-dataset analysis of the transcriptomes of thymic T cell populations (in vivo signal; GSE15907) and those of TCR-signaled peripheral CD4+ T cells data (in vitro signal; GSE48210). CCA was performed to identify the candidate genes used by both thymic and peripheral T cells. **(B)** Thymic T cell population data were analyzed by CCA using activated and resting T cells from GSE48210 as the explanatory variable. The output of CCA is composed of (A) cell sample score, (B) gene score, and (C) biplot value for explanatory variable, thus allowing the cross-level analysis of cells, genes, and biological process. Using the axis that is correlated with T cell activation, T cell activation scores of thymic T cell populations were specified and visualized. **(C)** Scatter plot showing the fold change increase by anti-CD3 stimulation and the correlation to the thymic T cell populations that received TCR signals. Cross-dataset analysis was performed by CCA of the transcriptome data from thymic T cell populations (in vivo signal; GSE15907) and peripheral CD4+ T cells that received in vitro anti-CD3 stimulation (in vitro signal; GSE48210) to identify candidate genes up-regulated by TCR signals in both thymic and peripheral T cells. **(D)** Time-course analysis of *Nr4a3* transcripts upon anti-CD3 stimulation (from GSE48210).

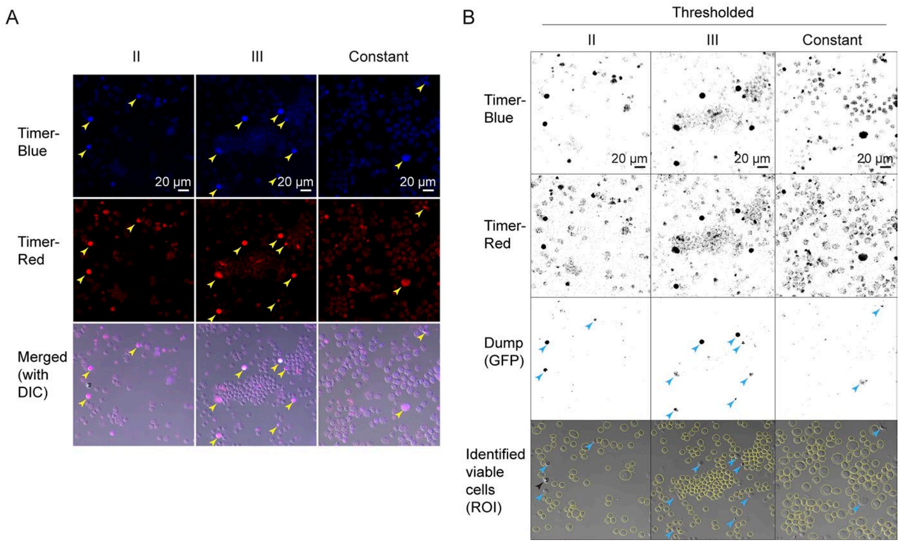

Figure S2. **Confocal microscopy analysis of OT-II *Nr4a3*-Tocky T cells stimulated at various frequencies. (A and B)** T cells from OT-II *Nr4a3*-Tocky mice were stimulated with various frequencies. Groups II and III received 4-h stimulation by Ova peptide within 48 h two and three times, respectively, and the constant group received Ova peptide stimulation continuously throughout the culture (see Fig. 4). Cells were attached to slides by Cytospin, fixed by paraformaldehyde, and immediately analyzed by confocal microscopy. **(A)** Confocal microscopy image of Timer–blue and Timer–red fluorescence as well as merged image (i.e., Timer–blue and Timer–red fluorescence with differential interference contrast [DIC]). Arrowheads indicate apoptotic cells. **(B)** The images in A were thresholded using the same value for all the samples from the same channel. The GFP channel was used as a dump data for identifying apoptotic cells as those with high autofluorescence. Differential interference contrast images were used to identify cells as region of interest (ROI), and the measurements in individual cells were redirected to thresholded grayscale images. Arrowheads indicate apoptotic cells identified by the dump GFP channel (colored) or the morphology (black).

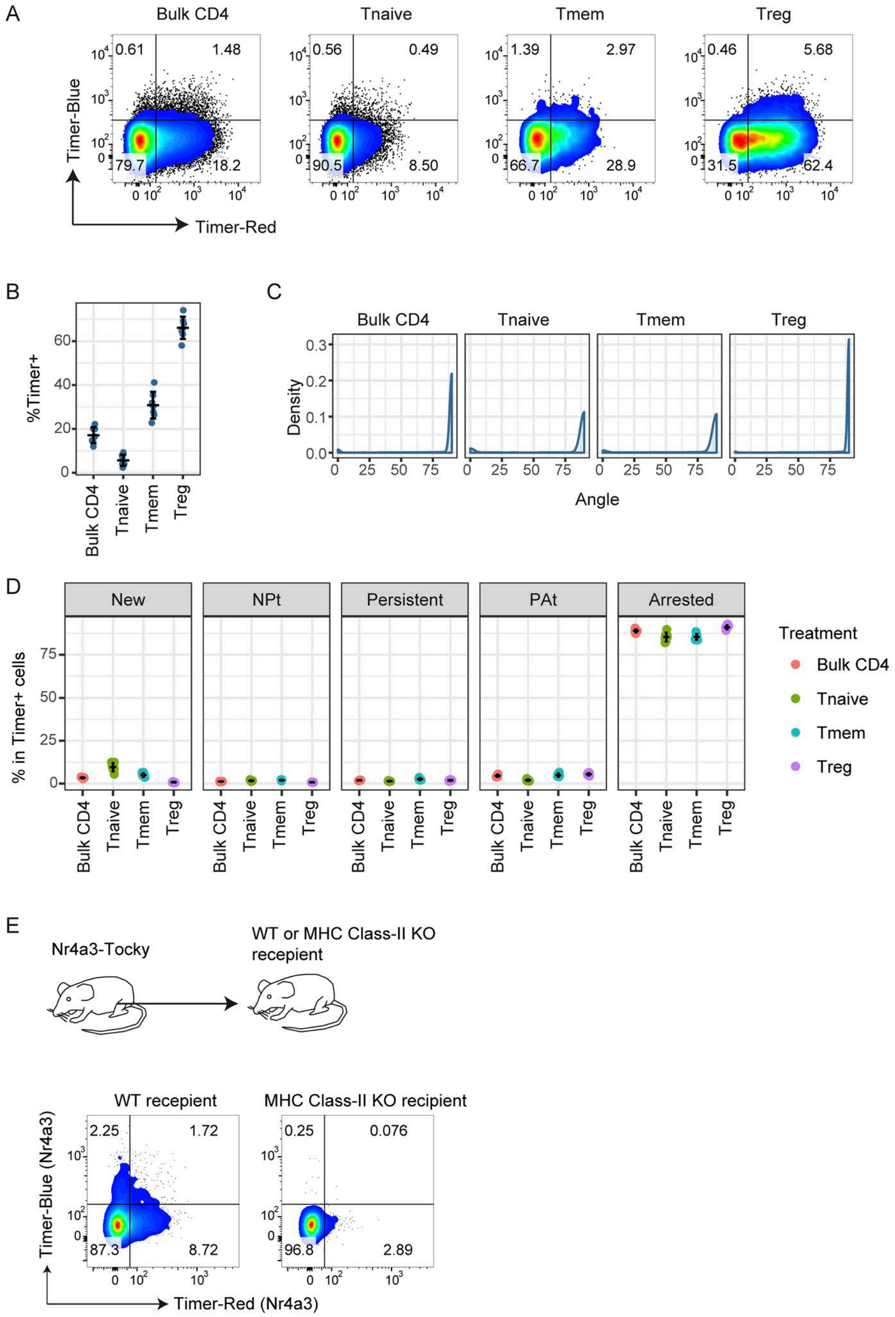

Figure S3. **Steady-state TCR signaling is restricted to memory-like T cells in vivo and dependent on MHC class II. (A–E)** *Nr4a3*-Tocky mice (polyclonal repertoire) were analyzed for Timer–blue versus Timer–red fluorescence in CD4$^+$ T cells. **(A)** Flow cytometry plots showing Timer blue versus Timer red fluorescence in the indicated populations from mesenteric lymph nodes. Bulk T cells showed majority of Timer-positive cells as red$^+$blue$^-$, meaning TCR signaling is relatively infrequent. CD4$^+$ T cells were sub divided into naive (CD4$^+$CD44^{lo}CD25$^-$Foxp3$^-$), memory (CD4$^+$CD44^{hi}CD25$^-$Foxp3$^-$), or Treg cells (CD4$^+$Foxp3$^+$). **(B)** Percentage Timer-positive cells in the four T cell subsets. **(C)** Density plot analysis reveals similar distribution of Timer Angles irrespective of CD4$^+$ T cell subset. **(D)** Timer locus analysis reveals that Treg cells are greatly enriched with recent TCR-signaled cells. n = 7; error bars represent mean ± SD. **(E)** CD4$^+$ T cells from *Nr4a3*-Tocky mice were adoptively transferred in to congenic WT or MHC class II knockout (I-Ab KO) mice. 9 d later, transferred T cells in the dLNs were analyzed for Timer–blue versus Timer–red fluorescence expression. 2–5% of T cells were receiving tonic signals in vivo, the majority of which were pure blue or pure red, indicating pulsatile signaling dynamics. Absence of Timer-positive cells in MHC class II knockout mice demonstrates that *Nr4a3*-Tocky signaling is dependent on pMHC–TCR interactions in vivo.

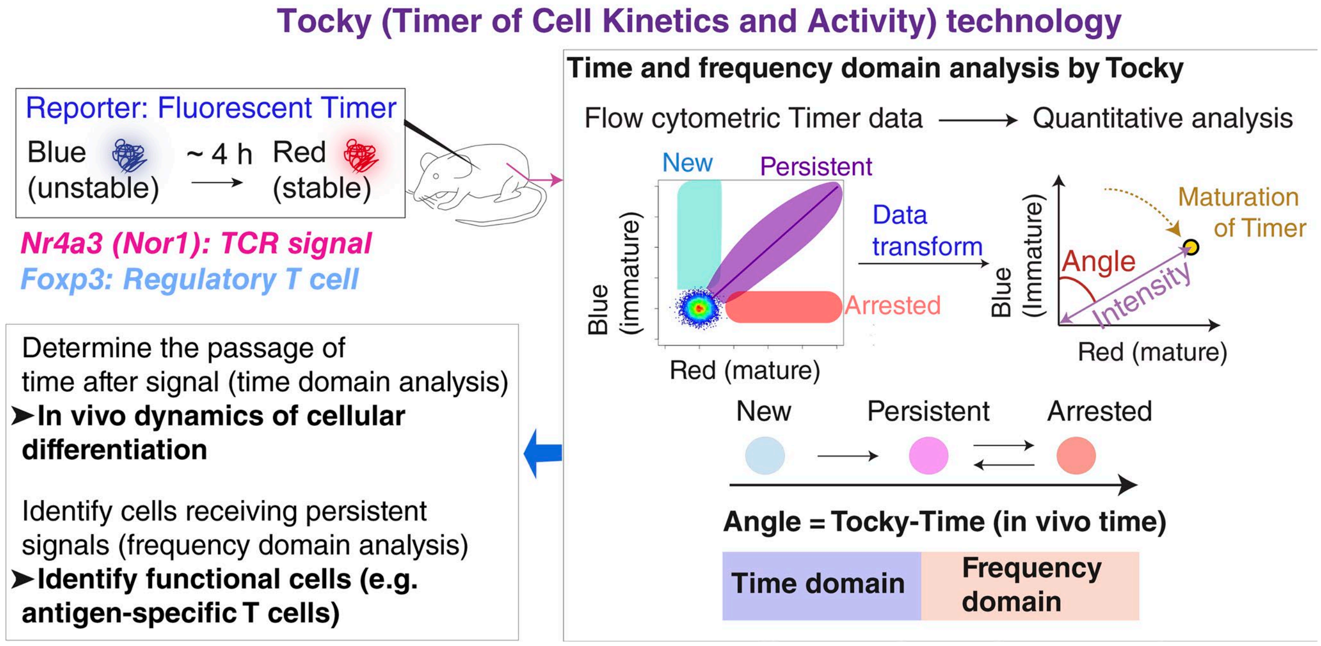

Figure S4. **Summary of Tocky technology.** Tocky system harnesses the power of fluorescent Timer protein in combination with a computational algorithm to define the Time and frequency domains of cellular differentiation (Tocky Time) using flow cytometry. This permits the determination of the relative passage of time after signaling cues as well as identifying cells that are receiving continuous or very frequent differentiation signals.

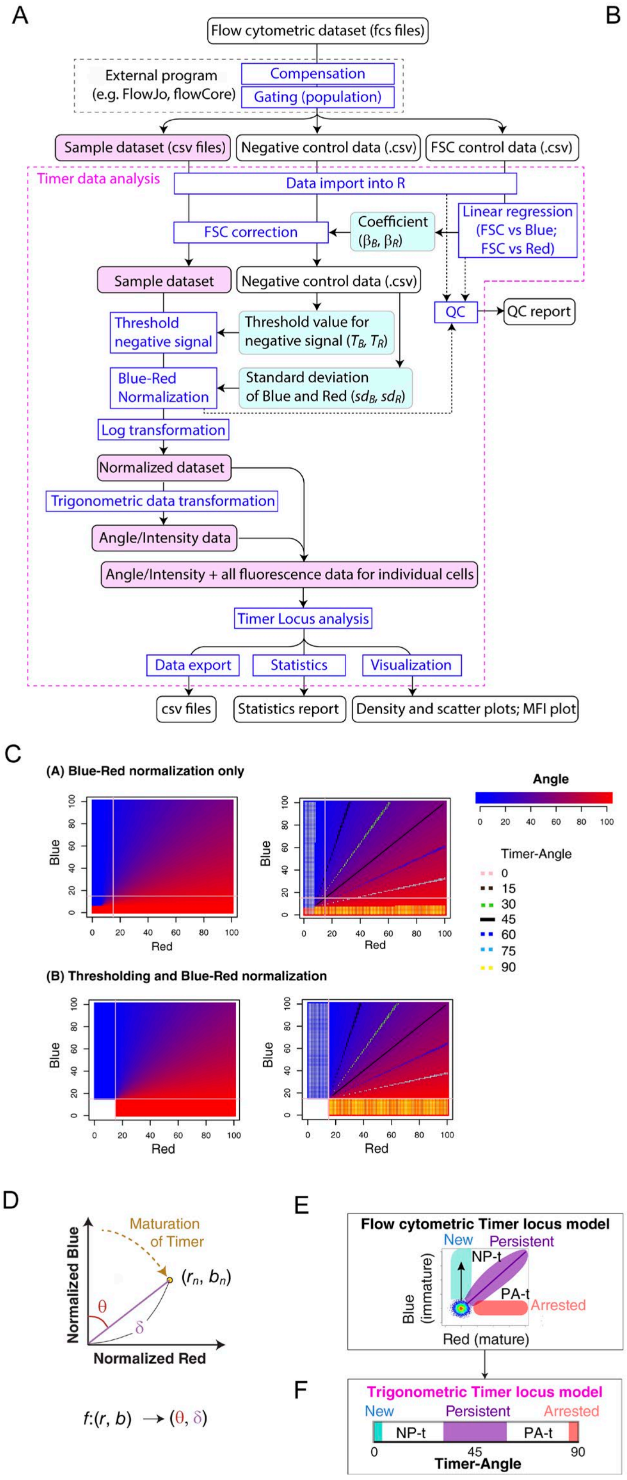

Figure S5. **Timer data analysis. (A)** Flow diagram detailing major components of Timer data analysis. MFI, mean fluorescence intensity. **(B)** Flow cytometric plots showing linear regression of Timer blue or Timer red versus FSC. Bottom plots show Timer blue or Timer red after correction for FSC. **(C)** Timer Angle calculation with or without data thresholding are compared on uniformly distributed cell data. Here, it is assumed that each cell has a discrete integer value between 0 and 100 for blue and another value for red (i.e., there are 10,201 cells in the blue–red plane) and that the barycenter of negative control cells is (10, 10). When data were processed by blue–red normalization without applying data thresholding, cells can have various Timer Angle values around the barycenter of negative control cells, and thus, cells in the negative quadrant gate (the lower left quadrant of pink lines) can have all the different Timer Angle values (top left) and have all the Timer loci (top right). These variations in blue$^-$red$^-$ cells are biologically meaningless. Data were first thresholded (at blue = 18 and red = 18 in this example; bottom left), and thereafter blue and red data were normalized. The thresholding removes all the blue$^-$red$^-$ cells. All the blue$^-$red$^+$ cells have a Timer Angle value of 90, whereas all the blue$^+$red$^-$ cells have a Timer Angle value of 0. **(D)** Calculation of Timer Angle based on normalized blue and red fluorescence. **(E)** Timer locus model applied to a theoretical flow cytometric plot of blue versus red Timer fluorescence. **(F)** The five Timer loci are designated as follows: New, 0°; NP-t, (0°, 30°); Persistent, (30°, 60°); PA-t, (60°, 90°); and Arrested, 90°. QC, quality control.

Table S1. **Differences between Tocky, fate-mapper, and GFP/FP reporters**

Analysis type	GFP reporter (and other conventional FP reporters)	Fate-mapping reporter (e.g., Cre: Rosa-FP double transgenic)	Tocky
Time domain analysis	No, do not distinguish new expressers from preexisting expressers	Not applicable	Reveal the time frame of early differentiation following key signaling
Frequency domain analysis	No, because of long half-life of reporter proteins	No information	Identification of cells with different frequencies of transcriptional activity and finding unique functional states (e.g., antigen-specific T cells)
